# Differential Responses of the Catalytic Efficiency of Ammonia and Nitrite Oxidation to Changes in Temperature

**DOI:** 10.3389/fmicb.2022.817986

**Published:** 2022-05-10

**Authors:** Anne E. Taylor, Brett L. Mellbye

**Affiliations:** ^1^Department of Crop and Soil Science, Oregon State University, Corvallis, OR, United States; ^2^Department of Microbiology, Oregon State University, Corvallis, OR, United States

**Keywords:** kinetics, substrate affinity, NH_3_ oxidation, NO_2_^–^ oxidation, catalytic efficiency, nitrification

## Abstract

Microbially mediated nitrification plays an important role in the nitrogen (N) cycle, and rates of activity have been shown to change significantly with temperature. Despite this, the substrate affinities of nitrifying bacteria and archaea have not been comprehensively measured and are often assumed to be static in mathematical models of environmental systems. In this study, we measured the oxidation kinetics of ammonia- (NH_3_) oxidizing archaea (AOA), NH_3_-oxidizing bacteria (AOB), and two distinct groups of nitrite (NO_2_^–^)-oxidizing bacteria (NOB), of the genera *Nitrobacter* and *Nitrospira*, by measuring the maximum rates of apparent activity (*V*_*max(app*)_), the apparent half-saturation constant (*K*_*m*(app)_), and the overall catalytic efficiency (*V*_*max(app)*_/*K*_*m*(app)_) over a range of temperatures. Changes in *V*_*max(app)*_ and *K*_*m*(app)_ with temperature were different between groups, with *V*_*max(app)*_ and catalytic efficiency increasing with temperature in AOA, while *V*_*max(app)*_, *K*_*m*(app)_, and catalytic efficiency increased in AOB. In *Nitrobacter* NOB, *V*_*max(app)*_ and *K*_*m*(app)_ increased, but catalytic efficiency decreased significantly with temperature. *Nitrospira* NOB were variable, but *V*_*max(app)*_ increased while catalytic efficiency and *K*_*m*(app)_ remained relatively unchanged. Michaelis–Menten (MM) and Haldane (H) kinetic models of NH_3_ oxidation and NO_2_^–^ oxidation based on the collected data correctly predict nitrification potential in some soil incubation experiments, but not others. Despite previous observations of coupled nitrification in many natural systems, our results demonstrate significant differences in response to temperature strategies between the different groups of nitrifiers; and indicate the need to further investigate the response of nitrifiers to environmental changes.

## Introduction

Anthropogenic activities have approximately doubled inorganic nitrogen (N) inputs to the global biosphere (Steffen et al., 2015), drastically altering nutrient cycling as evidenced by major changes in N utilization (Butterbach-Bahl et al., 2013; Liang et al., 2016) and carbon (C) sequestration (Davidson and Janssens, 2006). These changes have harmful effects on human and environmental health and severe economic impacts, including eutrophication of waterways (Shelton et al., 2018), significant changes in the sources and sinks of N oxide gas pollutants (Ni and Groffman, 2018), and the loss of biodiversity (Stevens, 2019). Nutrient cycling is microbially mediated by microorganisms that mineralize organic N to ammonium/ammonia (NH_4_^+^/NH_3_), oxidize NH_3_ to nitrite (NO_2_^–^) then nitrate (NO_3_^–^), and denitrify to N oxide and dinitrogen gases. Nitrification, the aerobic sequential oxidation of NH_3_ to NO_2_^–^ and then to NO_3_^–^, is an important intermediate in the N cycle and contributes to N availability for plants, N oxide gas emissions, and NO_2_^–^ and NO_3_^–^ leaching that leads to eutrophication (Galloway et al., 2008; Ward, 2011).

While environmental change predictions and climate models have focused on plant effects and C sequestration, the evaluation of environmental changes on microbial physiology has lagged behind or simply been treated as a “black box” (Shade et al., 2009; McGuire and Treseder, 2010; Treseder et al., 2012; Bennett et al., 2019). Nitrifying microorganisms are an important group that controls the size and composition of inorganic N pools available to plants (Ward, 2011). There is a critical need to understand the physiological changes that N cycling microbes make in response to climate change, and whether their responses can sustain environmental functionality and resilience. Nitrification is carried out by diverse microorganisms, including bacteria and archaea. NH_3_-oxidizing bacteria and archaea (AOB and AOA, respectively) generally carry out NH_3_ oxidation in partnership with NO_2_^–^-oxidizing bacteria (NOB; Daims et al., 2011; Hidetoshi et al., 2011; Sayavedra-Soto and Arp, 2011; Starkenburg et al., 2011). There are several groups of NOB, but in this study we focused on two groups that are the most abundant in soils: the genus *Nitrobacter* and the genus *Nitrospira*. A subset of lineage II *Nitrospira* that has the capability to completely oxidize NH_3_ to NO_3_^–^ (comammox) has not been included in this study (Ward, 2011; Daims et al., 2015; van Kessel et al., 2015).

Rates of biological enzymatic functions are temperature sensitive and there is evidence of differential responses of soil processes to temperature (Hobbs et al., 2013; Blagodatskaya et al., 2016; Taylor et al., 2017, 2019). Enzyme catalytic rates generally increase with temperature until an optimum is reached and, at higher temperatures, the reaction rate decreases due to thermal inactivation (Laidler and Peterman, 1979). Previous work by our research group and others suggests that AOB and AOA in soils respond differently to temperature (Pierre et al., 2017; Taylor et al., 2017; Duan et al., 2018), and it has recently shown that rates of activity and substrate affinity of soil NO_2_^–^ oxidation are also temperature-sensitive (Taylor et al., 2019; Duan et al., 2020). Increasing temperature can also increase reaction rates which are associated with increased production of N oxide, nitric oxide (NO), and nitrous oxide (N_2_O) gases (Law et al., 2012; Schreiber et al., 2012; Bonnett et al., 2013; Mellbye et al., 2016). However, increasing temperature can also result in lower affinity for the substrate, which may offset higher reaction rates (Laidler and Peterman, 1979; Snider et al., 2000; Davidson and Janssens, 2006; Blagodatskaya et al., 2016). Different responses to temperature by associated NH_3_ oxidizers and NO_2_^–^ oxidizers may affect nitrification coupling and potential NO_2_^–^ accumulation (Schaefert and Hollibaugh, 2017; Taylor et al., 2019; Duan et al., 2020; Venterea et al., 2020), but significant biochemical data, particularly *V*_*max*(app)_ and *K*_*m*(app)_, to model the response of nitrifying microorganisms to temperature are lacking.

Most physiological data on nitrifying bacteria and archaea have been generated under optimal growth conditions. For example, Suzuki et al. (1974) showed the response of *Nitrosomonas europaea K*_*m*(app)_ to changes in pH, but did not investigate temperature, and NH_3_ affinity has been measured in the AOBs *Nitrosococcus oceanus* and *Nitrosospira briensis*, but only at optimum temperature (Ward, 1987; Bollmann et al., 2005). The NO_2_^–^ oxidation kinetics of strains of *Nitrobacter* and *Nitrospira* have been determined only at each strain’s optimal growth temperature, pH, and dO_2_ (Nowka et al., 2015; Ushiki et al., 2017). Exceptions include a study measuring whole-cell kinetics of *Nitrosomonas* sp. 4W30 at three temperatures, which showed significantly greater affinity for NH_3_ at lower temperatures (Jones and Morita, 1985), and the evaluation of the AOA “*Ca. N. oleophilus*” MY3 kinetic response where the apparent *K*_*m*(app)_ increased ∼2-fold from 25 to 35^°^C (Jung et al., 2022).

Previous work demonstrated that the rates of nitrification changed with temperature (Kim et al., 2006; Schaefert and Hollibaugh, 2017; Taylor et al., 2017, 2019; Duan et al., 2018); however, it is unclear if changes in rates in response to temperature results from a shift in community structure or changes in microbial physiology (Blagodatskaya et al., 2016; Taylor et al., 2017; Duan et al., 2020). We hypothesized that individual nitrifiers respond differently to temperature, and that these responses are reflected in changes in substrate affinity and reaction rate. We chose pure culture representative members of AOA, AOB, *Nitrobacter* NOB, and *Nitrospira* NOB for evaluation and investigated changes in NH_3_ and NO_2_^–^ affinity and oxidation rate over a large range of physiologically relevant temperatures. Finally, we applied Michaelis–Menten (MM) and Haldane (H) kinetic models, based on observations, to changes in nitrification rates observed in whole soils over a range of temperatures.

## Materials and Methods

### Nitrifying Strains and Growth Conditions

Nine nitrifying microorganisms were used in this study and cultivated as previously described (Table 1). We utilized the AOA *Nitrososphaera viennensis* (Tourna et al., 2011; Taylor et al., 2015), and the AOB *N. europaea* and *Nitrosospira multiformis* (Ensign et al., 1993; Chain et al., 2003; Norton et al., 2008; Taylor et al., 2013). We worked with a total of six NOB strains. *Nitrobacter hamburgensis*, *Nitrobacter winogradskyi*, *Nitrobacter vulgaris*, and *Nitrospira moscoviensis* were grown under the conditions previously used in our laboratories (Bock et al., 1983, 1990; Ehrich et al., 1995; Starkenburg et al., 2006, 2008; Koch et al., 2015; Mellbye et al., 2017a,b), and *Nitrospira japonica* NJ1 and *Nitrospira* ND1 were cultivated as previously described (Ushiki et al., 2017). Minimal media for all cultures were at pH 8. Cultures were grown at their optimal growth temperatures (30^°^C for all cultures except for *N. viennensis*, which was grown at 42^°^C and *N. moscoviensis* which was grown at 37^°^C, Table 1) to maximize growth yields and for the ease of comparison with other studies. Cultures were monitored at regular intervals to check cell density by optical density at 600 nm (OD_600_) and NO_2_^–^ concentrations by the Griess assay.

**TABLE 1:** Ammonia (NH_3_) and nitrite (NO_2_^–^) oxidizer strains for this study were grown under optimal growth conditions and temperatures*^a^*.

Genus	Species	AOA/AOB/NOB*^b^*	Strain designation	Growth temperature (^°^C)
*Nitrososphaera*	*viennensis*	AOA	EN76	42
*Nitrosospira*	*multiformis*	AOB	ATCC 25196(T)	30
*Nitrosomonas*	*europaea*	AOB	ATCC 19718	30
*Nitrobacter*	*winogradskyi*	NOB	NB-255	30
*Nitrobacter*	*hamburgensis*	NOB	X14	30
*Nitrobacter*	*vulgaris*	NOB	AB_1_	30
*Nitrospira*	*moscoviensis*	NOB	M1	37
*Nitrospira*	*japonica*	NOB	NJ1	30
*Nitrospira*	sp.	NOB	ND1	30

*^a^Bock et al. (1983, 1990), Ehrich et al. (1995), Chain et al. (2003), Starkenburg et al. (2006, 2008), Norton et al. (2008), Tourna et al. (2011), Koch et al. (2015), Mellbye et al. (2017b), and Ushiki et al. (2017). ^b^NH_3_-oxidizing archaean, bacterium, or NO_2_^–^-oxidizing bacterium.*

### Determination of Kinetic Parameters

Cultures were grown to early stationary phase and then harvested by centrifugation (10,000 × *g*, 15 min), washed with their respective substrate-free growth media to remove trace concentrations of NH_3_ and NO_2_^–^, and resuspended in ∼15-ml volumes of substrate-free growth media (pH 8) to yield a concentrated cell suspension. The cell density of the concentrated suspension was measured by OD_600_, and the protein content was measured with a Pierce bicinchoninic acid protein assay kit (Thermo Scientific, Rockford, IL, United States) to determine the volume of cells to be added to the incubation vials, yielding an average protein concentration of 2.6 ± 2.9 μg/μl for the NOB and 6.5 ± 1.7 μg/μl for NH_3_ oxidizers. For each experimental temperature, vials were prepared in triplicate with 5 ml portions of their respective growth media (pH 8) supplemented with a range of substrate concentrations (NH_4_^+^ for the AOA and AOB and NO_2_^–^ for the NOB). The vials were closed with gray butyl stoppers and preincubated at the experimental temperatures. Experiments were initiated with the addition of aliquots of concentrated culture. Vials were sampled immediately and then at ∼30 min intervals to obtain NO_2_^–^ concentration at five time points over 2 h, with a final time point obtained at ∼4 h after the initiation of the experiment. Rates of NH_3_ oxidation by the AOA and AOB were determined by the rate of NO_2_^–^ accumulation in the incubation vials, and NO_2_^–^ oxidation rates by its disappearance. The MM kinetic coefficients of substrate utilization at each temperature were determined by fitting the NO_2_^–^ or NH_3_ oxidation rate (*v*) for a given substrate concentration (*S*) to the equation


(1) $$v=\frac{V_{\text{max}}\times S}{K_{m}+S}$$

to find the maximum reaction rate (*V*_*max*_) and the substrate concentration that yields half of this maximum rate (*K*_*m*_) using SigmaPlot (Systat Software, San Jose, CA, United States). Because we are working with whole cells, the calculated *V*_*max*_ and *K*_*m*_ should be considered to be the apparent *V*_*max*_ (*V*_*max(app*)_) and apparent *K*_*m*_ (*K*_*m*(app)_). Note that we calculated *V*_*max*(app)_ as a relative *k*_*cat*_ based on the maximum reaction rate per mg protein of whole cells (Snider et al., 2000). The effects of H inhibition on substrate utilization were determined by fitting the NO_2_^–^ or NH_3_ oxidation rate for a given substrate concentration to the equation


(2) $$v=\frac{V_{\text{max}}\times S}{\left(K_{m}+S+\frac{S^{2}}{K_{i}}\right)}$$

to find the effects of substrate inhibition on *V*_*max(app)*_ and *K*_*m*(app)_, as well as to find the substrate concentration that inhibited *V*_*max(app)*_ by 50% (*K*_*i*_). Specific affinity for substrate (*a*^o^*_*s*_*) and the affinity constant (*K*_*A*_) were determined as described by Button (1998). Temperature-dependent distribution of NH_3_/NH_4_^+^ was calculated as described by Groeneweg et al. (1994).

### Soil Incubations to Characterize NH_3_ Oxidation Response

Agricultural soils were collected from the western Oregon Coastal Plain in Tillamook County and from the eastern Oregon Columbia River basin in Morrow County (Waggoner et al., 2021). The western Oregon Coastal Plain soil is identified as a Quillamook series silt loam soil^1^. It receives a mean annual precipitation of 2,159 mm and has an isomesic temperature regime (temperatures averaging 6.5^°^C in winter and 14^°^C in summer) with a mean annual temperature of 10^°^C and has a pH of 5.8. The soil was cropped with a silage corn (***Zea mays*** L.) and rye grass (***Lolium*** spp.) rotation. The eastern Oregon Columbia River basin soil receives a mean annual precipitation of 178 mm and has a mesic temperature regime (temperatures averaging 2^°^C in winter to 22^°^C in summer) with a mean annual temperature of 12^°^C and has a pH of 8.5. It is identified as a Sagehill series sandy loam soil (see text footnote **1**). It is cropped under a silage corn (***Z. mays*** L.) and triticale (***Triticosecale***) rotation. Three field replicates were collected at each site, each of which was created using a composite of 3–5 samples from the surface 0–20 cm that were mixed in the field, and then brought to the laboratory where they were sieved at 2.36 mm and stored at 4^°^C until use.

Using a method previously adapted by DeAngelis et al. (2010), DNA extraction was performed using phenol:chloroform:isopropyl alcohol (25:24:1), followed by precipitation in PEG6000 solution [30% (w/v) polyethylene glycol 6000 in 1.6 M NaCl] (DeAngelis et al., 2010). Each soil sample was extracted two times per replicate, with three replicates. Extractions from each replicate were pooled. Quantitative polymerase chain reaction (qPCR) was then used to determine gene copy numbers of AOA *amoA*, AOB *amoA*, and NOB *nxrA* and *nxrB* using reagents, primers, and conditions as described in Supplementary Table 1. Standards for qPCR were prepared from *N. viennensis* (AOA *amoA*), *N. multiformis* (AOB *amoA*), *N. winogradskyi* (*nxrA*), and *N. moscoviensis* (*nxrB*).

Soil incubations were performed to evaluate the contributions of AOA and AOB to soil nitrification when NH_4_^+^ was supplied as NH_4_Cl. Addition of 1-octyne (4 μM) allows differentiation of AOA and AOB through inactivation of the AOB NH_3_ monooxygenase (AMO), and additions of acetylene (10 μM) were used to prevent all NH_4_^+^-mediated nitrification of both AOA and AOB, serving as a negative control (Taylor et al., 2013, 2015). To evaluate the effect of temperature on NH_3_ response, incubations were established at a range representative of temperatures during the growing season at each sampling site, plus one temperature 10^°^C higher than normal temperatures to evaluate the effect of temperature stress. The incubation temperatures for Coastal Plain soils were 10, 20, and 30^°^C, while Columbia Basin soils were incubated at 10, 20, 30, and 40^°^C. Prior to experimentation, three field replicates of each soil were leached to remove background NO_3_^–^ to increase colorimetric assay sensitivity. Briefly, soil aliquots were placed in a funnel lined with a paper filter and covered with crushed ice, then held at room temperature while the ice melted and percolated through the soil. This leaching method allows the soil to retain most of its structure. Soils were then allowed to partially air dry to ∼30% gravimetric moisture content for the Coastal Plain and 5% for the Columbia Basin. Leached soils (2.5 *g*) were measured into 125-ml Wheaton bottles and preincubated at room temperature (23^°^C) for 12–24 h before experimentation.

To initiate the incubations, soils received 5 μmol NH_4_Cl g^–1^ soil in dH_2_O to bring the soil moisture content slightly above the field capacity for each soil (72% for the Coastal Plain and 22% for the Columbia Basin). A control that received dH_2_O, but no NH_4_^+^ source, was included for each treatment. Each bottle was capped using a lid with an *n*-butyl septum and alkyne treatments imposed as described above. Alkyne stocks were prepared and added in gaseous forms as previously described to the +octyne and +acetylene treatments (Giguere et al., 2015). The soils were then incubated in the dark for 24 h at 10, 20, 30, or 40^°^C.

At the end of the incubations, the accumulations of NO_2_^–^ and NO_2_^–^ + NO_3_^–^ in soils were determined as previously described (Miranda et al., 2001; Hood-Nowotny et al., 2010). The difference in NO_2_^–^ or NO_2_^–^ + NO_3_^–^ compared to incubations that included acetylene was considered to represent the rate of nitrification performed by the nitrifiers, or total AOA and AOB activity. The difference between +octyne treatments and +acetylene treatments represents only AOA activity. AOB activity was then calculated as the difference between total nitrification activity and AOA-only activity. NOB was considered to be equal to the total rate of soil nitrification minus any accumulated NO_2_^–^.

### Modeling the Potential Soil Response of AOA, AOB, and NOB Cultures

#### Coastal Plain Soil

To determine whether MM kinetics and H (substrate-induced inhibition) parameters could be used to predict the rates of NH_3_ and NO_2_^–^ oxidation activity in soil incubations, certain assumptions were made to apply the rates from cultures (μmol substrate mg protein^–1^ h^–1^) to soils in which population size was determined by qPCR of AMO and NO_2_^–^ oxidoreductase (NXR) genes (Supplementary Table 1), and the rates were expressed as μmol substrate g soil^–1^ h^–1^. Because the qPCR primers did not discriminate between the strains of AOB or NOB, when evaluating the potential soil activity of each of the cultures we assumed that each strain made up the entire population. It was also assumed that all gene copy numbers determined through qPCR came from active cells, and this number was divided by the number of gene copies per genome as determined by the Basic Local Alignment Search Tool (BLAST; Supplementary Table 2). From Urakawa et al. (2011), the relationship between cell volume and cell protein content of *Nitrosopumilus maritimus* has been determined, and we extrapolated that information to estimate the protein content per cell of the cultures used in this study (Supplementary Table 3); and in turn estimate the amount of cell protein in each gram of soil (see Supplementary Material for further explanation on the conversion of μmol substrate g soil^–1^ h^–1^ to μmol substrate g soil^–1^ h^–1^).

It was also necessary to make assumptions about starting substrate concentrations in the soil. The Coastal Plain soil without NH_4_^+^ addition had 16 μmol g soil^–1^ KCl extractable NH_3_ + NH_4_^+^ and a soil water content of 72%, and the assumption was made that all extractable NH_4_^+^ would be in soil solution, yielding a concentration of 0.22 μM NH_3_ + NH_4_^+^. In the incubations with NH_4_^+^ addition (5 μmol g soil^–1^), it was assumed that all NH_4_^+^ would be in the soil solution yielding a concentration of 6 mM NH_3_ + NH. To evaluate how the rates of MM or H kinetics of NOB cultures would respond under soil conditions, it was necessary to estimate possible concentrations of NO_2_^–^ in the soil. Our confident limit of detection of NO_2_^–^ in soil is ≥2 μM NO_2_^–^. There was no detectable NO_2_^–^ in Coastal Plain soils prior to, or during incubations, suggesting that the rate of soil NOB activity was equal to the rate of soil NH_3_ oxidation; but does not rule out the possibility that trace NO_2_^–^ accumulated during active soil nitrification. For the Coastal Plain soil without NH_4_^+^ addition, there was 0.22 μM extractable NH_3_ + NH_4_^+^ and, if completely oxidized to NO_2_^–^, would yield 0.22 μM NO_2_^–^; this value was used as the substrate concentration. For Coastal Plain soils with NH_4_^+^ addition, we assumed a NO_2_^–^ concentration just below our level of detection (2 μM NO_2_^–^).

The above assumptions were used with the kinetic parameters determined in this study (Supplementary Table 5) for each of the microbial strains to estimate the potential rate of activity in the soil using MM Eq. (1) and H Eq. (2) kinetics at each soil incubation temperature. The average value of population size, cell protein content, gene copy number, *V*_*max(app)*_, *K*_*m*(app)_, and *K*_*i*(app)_ were used in the calculations as there were an excessive number of potential combinations (2^6^ or 64 combinations at each temperature) of these values if both extremes of the standard deviation (SD) were evaluated. We assumed no inhibition of NH_3_ oxidation in the incubations without NH_4_^+^ addition. Additionally, H kinetics were not determined for NOB in the treatments without NH_4_^+^ addition where NO_2_^–^ was assumed to be 0.22 μM and several orders of magnitude lower than *K*_*i*_ determined in this study.

#### Columbia Basin Soil

The assumptions made on the population size were the same as for the Coastal Plain soil detailed above. The Columbia Basin soil without NH_4_^+^ addition had 0.01 μmol g soil^–1^ KCl extractable NH_3_ + NH_4_^+^ and a soil water content of 22%, and the assumption was made that all extractable NH_4_^+^ would be in the soil solution yielding a concentration of 0.05 μM NH_3_ + NH_4_^+^. In the incubations with NH_4_^+^ addition (5 μmol g soil^–1^), it was assumed that all NH_4_^+^ would be in the soil solution yielding a concentration of 22 mM NH_3_ + NH. There was no detectable NO_2_^–^ in the Columbia Basin soil prior to incubation or in the incubations without NH_4_^+^ addition, suggesting that the rate of soil NOB activity was equal to the total rate of soil nitrification. In the Columbia Basin soil incubations without NH_4_^+^ addition, we assumed a NO_2_^–^ concentration of 0.05 μM (all extractable soil NH_4_^+^ oxidized to NO_2_^–^). However, in the Columbia Basin soils with NH_4_^+^ addition, NO_2_^–^ accumulated during incubations to concentrations of 2, 700, 1,900, and 400 μM at 10, 20, 30, and 40^°^C, respectively; and these values were used in the calculation of MM and H kinetic rates. In the treatments with NH_4_^+^ addition in which NO_2_^–^ accumulated, the rate of NO_2_^–^ oxidation was the NH_3_ oxidation rate minus the rate of accumulated NO_2_^–^.

### Statistical Analysis

Analysis of variance (ANOVA) using the Holm–Sidak method in SigmaPlot (Systat Software, San Jose, CA, United States) was used to evaluate if there were significant differences in kinetic parameters across temperatures. Regression analyses in SigmaPlot compared the MM and H kinetic models with the rates of NH_3_ and NO_2_^–^ oxidation measured in Coastal Plain and Columbia Basin soils.

### Bioinformatic Analyses

The National Center for Biotechnology Information^2^, Kyoto Encyclopedia of Genes and Genomes^3^, and BLAST^4^ were used to identify AmoA and NxrA homologs in nitrifiers and other bacteria (Supplementary Datasets 1, 2; Boratyn et al., 2013; Kanehisa et al., 2017). MUSCLE was used for multiple alignment of amino acid sequences (Edgar, 2004), and phylogenetic analyses were conducted using MEGA11 (Kumar et al., 2016). The evolutionary history of AmoA and NxrA was inferred using the Maximum Likelihood method, based on the JTT matrix model (Jones et al., 1992). Phylogenetic trees were visualized using FigTree 1.4.4^5^.

## Results

### Homology of AmoA and NrxA in Nitrifying Bacteria and Archaea

**FIGURE 1:**
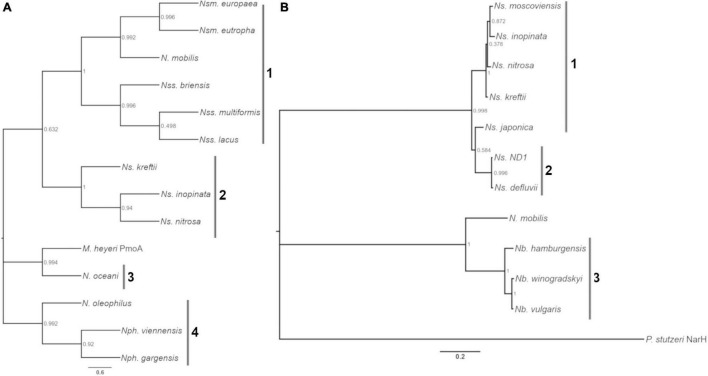
Evolutionary history of AmoA and NxrA. Phylogenetic trees of AmoA **(A)** and NxrA **(B)** homologs from nitrifiers and other bacteria. **(A)** Numbered bars indicate clades: 1, *Nitrosomonas* and *Nitrosospira*, 2, Comammox *Nitrospira*, 3, *Nitrosococcus*, and 4, AOA. **(B)** Numbered bars indicate clades: 1, *Nitrospira* lineage II, 2, *Nitrospira* Lineage I, and 3, *Nitrobacter*. The scale bar indicates the mean number of substitutions per site. Bootstrap values from 500 resamplings are rounded to two digits. The protein name of the homolog follows the genus and species, if not AmoA or NxrA. Strain designation and accession numbers can be found in Supplementary Datasets 1, 2.

Enzyme kinetics are notably different between AOB and AOA, and between *Nitrobacter* NOB and *Nitrospira* NOB; perhaps due to different niche specialization and temperature optima (Martens-Habbena et al., 2009; Verhamme et al., 2011; Prosser and Nicol, 2012; Pester et al., 2014; Gruber-Dorninger et al., 2015; Nowka et al., 2015; Kits et al., 2017; Ushiki et al., 2017). We chose to analyze the oxidation rate and affinity of nine nitrifying microorganisms isolated from soils and wastewater systems, and we focused on microorganisms that could accumulate the biomass necessary for our experiments (Table 1). For ammonia oxidizers (AOA and AOB), we selected the AOA *N. viennensis* and the AOB *N. multiformis* and *N. europaea*. The evolutionary history of the putative substrate-binding subunits of AMO (putative substrate-binding subunit, AmoA) is shown in Figure 1A. For NOB, *N. winogradskyi*, *N. hamburgensis*, *N. vulgaris, N. moscoviensis*, *N. japonica*, and *Ns.* ND1 were selected. The evolutionary history of the putative substrate-binding subunits of NXR (putative substrate-binding subunit, NxrA) is shown in Figure 1B.

### Nitrifier Kinetics Change With Temperature

**FIGURE 2:**
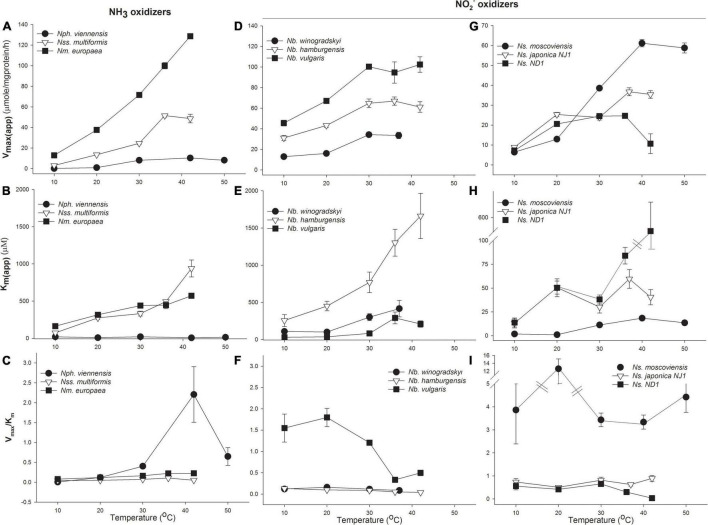
Response of ammonia (NH_3_) and nitrite (NO_2_^–^) oxidizers to temperature. Michaelis–Menten (MM) kinetic parameters for NH_3_-oxidizing bacteria and archaea (AOA, AOB; **A–C)**, *Nitrobacter* NO_2_^–^ oxidizing bacteria (NOB; **D–F)**, and *Nitrospira* NOB **(G–I)**. Cultures were grown at their optimal growth temperature, and *V*_*max(app)*_ and *K*_*m*(app)_ were determined at the indicated temperatures during short incubations. The response of NH_3_ oxidizers is expressed in terms of total NH_3_+NH_4_^+^; see Supplementary Table 6 for the response to NH_3_. Catalytic efficiency was calculated as *V*_*max*_*/K_*m*_*. Error bars represent the standard deviation (SD) of the average of three replicates.

The oxidation rates of nitrifiers and their affinity in pure culture incubations were investigated. Both MM and H kinetic models were used to determine *V*_*max(app)*_, *K*_*m*(app)_, and catalytic efficiency (V_*max*_*/K_*m*_*) from pure culture data (Supplementary Table 5). We have presented these data showing changes in *V*_*max(app)*_, *K*_*m*(app)_, and catalytic efficiency in Figure 2. As expected, *V*_*max(app)*_ increased with temperature in all microorganisms we investigated; however, there were significant differences in how enzyme affinity (*K*_*m*(app)_) changed with temperature between the different groups of nitrifiers.

Bacterial and archaeal NH_3_ oxidizers had significantly different changes in enzyme affinity with temperature. For the AOA representative, *N. viennensis, V*_*max(app)*_ increased ∼100-fold from 10 to 42^°^C, with maximum rates of 10.3 μmol substrate mg protein^–1^ h^–1^. *N. viennensis* had no significant change in *K*_*m*(app)_ with temperature, with values ranging from 6.1 to 20.3 μM NH_3_ + NH_4_^+^, suggesting that temperature generally does not significantly affect enzyme affinity but does increase catalytic efficiency (Figure 2A and Supplementary Tables 5, 6 compares the affinity of the NH_3_ oxidizers for total NH_3_ + NH_4_^+^ vs. NH_3_). There was evidence of temperature-independent H substrate-induced inhibition of *N. viennensis* at 0.3–2.4 mM NH_3_ + NH_4_^+^ (Supplementary Table 5). In contrast, the *K*_*m*(app)_ of AOB significantly increased with temperature from 10 to 37^°^C, suggesting that increasing temperature decreases bacterial AMO affinity; potentially explaining previous observations of different temperature optima between AOA and AOB (Figure 2B). In addition, while *K*_*m*(app)_ of NH_3_ oxidation for AOB increased 10- to 12.7-fold with temperature (from 73.7 to 938.6 μM NH_3_ + NH_4_^+^), the *V*_*max(app)*_ of AOB also increased 10- to –17-fold (from 51.5 to 128.7 μmol NH_3_ + NH_4_^+^ mg protein^–1^ h^–1^), resulting in increased catalytic efficiency. In both *N. viennensis* and *N. multiformis, V*_*max(app)*_ decreased when temperature increased past their temperature optima resulting in decreased catalytic efficiency (Figure 2C).

There were strikingly different kinetic responses of NO_2_^–^ oxidation to temperature between *Nitrobacter* and *Nitrospira* and also between the different lineages (Figures 2D–I and Supplementary Table 5). In *Nitrobacter*, both *V*_*max(app)*_ and *K*_*m*(app)_ increased significantly as temperature increased from 20 to 37^°^C (Figures 2D,E). While *V*_*max(app)*_ increased from ∼2- to 2.6-fold (from 33.6 to 102.4 μmol NO_2_^–^ mg protein^–1^ h^–1^), fold increases in *K*_*m*(app)_ were approximately two times that of *V*_*max(app)*_ (3.8–6.9-fold) and ranged from 416.5 to 1662 μM NO_2_^–^ as temperature increased; resulting in significantly decreased catalytic efficiency (Figure 2F and Supplementary Table 5).

There were interesting differences among the three *Nitrospira* strains tested. The lineage II *Nitrospira*, *N. moscoviensis* (growth temperature 37^°^C), and *N. japonica NJ1* (growth temperature 30^°^C), behaved similarly, except for notable differences in temperature optima that resulted in maximum *V*_*max(app)*_ at 37^°^C or 42^°^C for *N. moscoviensis* and *N. japonica NJ1*, respectively, (Figure 2G). There were also indications of H inhibition of *N. moscoviensis* that became most pronounced at 10^°^C (Supplementary Table 5). *V*_*max(app)*_ increased from 1.5- to 4.1-fold, to 19.9 to 35.4 μmol NO_2_^–^ mg protein^–1^ h^–1^ for lineage II *Nitrospira* while *K*_*m*(app)_ varied between 6.6 and 36.8 μM NO_2_^–^ with little relationship to temperature (Supplementary Table 5). Except for a ∼4-fold increase in catalytic efficiency for *N. moscoviensis* at 20^°^C, there was little difference in catalytic efficiency over the temperature ranges tested for the two evaluated lineage II *Nitrospira* (Figure 2I). The lineage I *Nitrospira* strain, *Ns.* ND1, had a 3.4-fold increase in *V*_*max*(app)_ up to 24.5 μmol NO_2_^–^ mg protein^–1^ h^–1^, and little change in *K*_*m*(app)_ (13.9 to 50.4 μM NO_2_^–^), resulting in small changes in catalytic efficiency from 10 to 30^°^C. However, from 30 to 42^°^C, *V*_*max*(app)_ stopped increasing and eventually decreased, and *K*_*m*(app)_ drastically increased to 505.9 μM, and catalytic efficiency significantly decreased (Figure 2I and Supplementary Table 5).

### Comparison of the Nitrification Temperature Response Predicted by Michaelis–Menten and Haldane Kinetic Models to Soil Nitrification Activity

**FIGURE 3:**
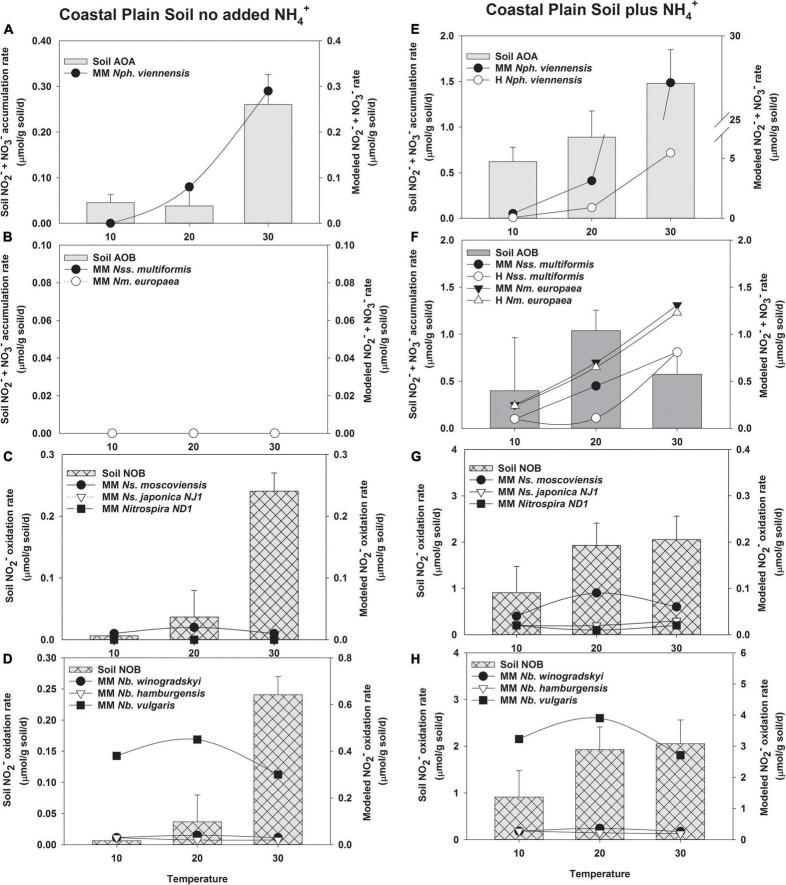
Comparison of the potential response of AOA, AOB, and NOB cultures evaluated with the response of soil nitrifiers in short-term (24 h) incubations of Coastal Plain soil without no NH_4_^+^ addition **(A–D)**, and with NH_4_^+^ addition (5 μmol/g soil, **E–H)**. The soil nitrifier response measured during the incubations is indicated by the bars corresponding to the left *y*-axis, and the response modeled using the kinetics of nitrifier cultures determined in this study (Supplementary Table 5) is indicated by the symbols corresponding to the right *y*-axis. Individual nitrifier names are preceded by either MM or Haldane (H) to indicate that MM or H parameters, respectively, were used to calculate the potential rates of activity.

**FIGURE 4:**
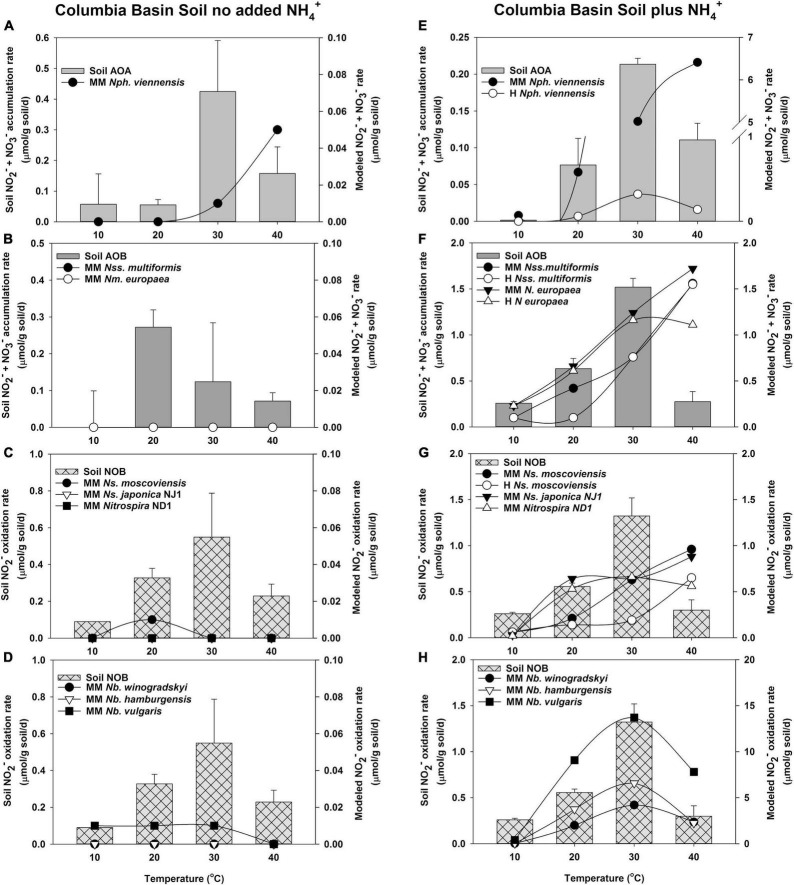
Comparison of the potential response of AOA, AOB, and NOB cultures evaluated with the response of soil nitrifiers in short-term (24 h) incubations of Columbia Basin soil with no NH_4_^+^ addition **(A–D)**, and with NH_4_^+^ addition (5 μmol/g soil, **E–H)**. The soil nitrifier response measured during incubations is indicated by the bars corresponding to the left *y*-axis, and the response modeled using the kinetics of nitrifier cultures determined in this study (Supplementary Table 5) is indicated by the symbols corresponding with the right *y*-axis. Individual nitrifier names are preceded by MM or H to indicate that MM or H parameters, respectively, were used to calculate the potential rates of activity.

After determining kinetic values for NH_3_ and NO_2_^–^ oxidation, we applied the MM and H kinetic models to the changes in nitrification rates observed in short-term incubations of whole soils with or without NH_4_^+^ addition and incubated at temperatures from 10 to 40^°^C (Figures 3, 4). We reasoned that this approach would yield insight into how well pure culture microorganisms used to make the model resemble naturally occurring nitrifying microorganisms. Agricultural soils from two climatic regions of Oregon that demonstrate different nitrification properties were used in this study. AOA outnumbered AOB in both soils (Supplementary Table 4). There was more *Nitrobacter* than *Nitrospira* NOB in Coastal Plain soils; however, there was no significant difference in these two populations in the Columbia Basin soil. Nitrification in Coastal Plain soils was coupled and NO_2_^–^ did not accumulate, while NO_2_^–^ accumulated in Columbia Basin soils despite having lower overall rates of NH_3_ oxidation (Figures 3, 4). H models include substrate inhibition and are expected to be important in AOA and *Nitrospira* NOB that prefer low concentrations of NH_3_ or NO_2_^–^, respectively.

### Model Predictions Compared to Nitrification in Coastal Plain Soils

Soil AOA had activity in Coastal Plain soils without NH_4_^+^ addition, and the NH_3_ oxidation rate predicted by the MM model was a good fit for soil AOA activity (*R*^2^ = 0.815, *p* = 0.01, Supplementary Table 7, and Figure 3A). Unlike AOA, no activity of soil AOB was measured in the absence of NH_4_^+^ addition in Coastal Plain soils, and the AOB MM models also predicted no contribution to NH_3_ oxidation by soil AOB (Figure 3B). In the absence of NH_4_^+^ addition, the MM model did not predict any significant response by *N. japonica NJ1* or *Nitrospira* ND1; however, the rate response of *N. moscoviensis* determined by the kinetic parameters of the MM model matched the data at 10 and 20^°^C, but not 30^°^C (Figure 3C). The rate response of *N. vulgaris* determined by the kinetic parameters of the MM model could potentially contribute to all the NO_2_^–^ oxidation observed in this soil (*R*^2^ = 0.641, *p* = 0.01), but the MM models for the remaining NOB do not predict any contribution to NO_2_^–^ oxidation (Figure 3D).

Addition of NH_4_^+^ to Coastal Plain soils increased the rates of NO_2_^–^ + NO_3_^–^ production by soil AOA ∼5–10-fold (Figure 3E). H substrate-induced inhibition of *N. viennensis* predicted a lower AOA contribution to NH_3_ oxidation than that observed in the soil at 10 and 20^°^C, although the potential response of AOA like *N. viennensis* exceeded the response observed in the soil at 30^°^C (*R*^2^ = 0.685, *p* = 0.006, Figure 3E). In the Coastal Plain soil with NH_4_^+^ addition, soil AOB contributed to nitrification at all three incubation temperatures, providing NH_3_ oxidation rates approximately equal to that of soil AOA at 10 and 20^°^C, and ∼25% of total NO_2_^–^ + NO_3_^–^ production at 30^°^C; however, neither the MM or H models for *N. multiformis* or *N. europaea* were a good fit to the data (Supplementary Table 7 and Figure 3F). The H model predicted no significant effects of substrate inhibition on *N. europaea* (data not shown) but predicted a reduction in *N. multiformis* contributions at 20^°^C. The MM models for *N. europaea* and *N. multiformis* showed increasing rates of NO_2_^–^ + NO_3_^–^ production as temperature increased from 10 to 30^°^C, whereas soil AOB rates were the highest at 20^°^C. The MM models predicted that AOB with kinetic characteristics like those of *N. europaea* and *N. multiformis* could contribute much of the NO_2_^–^ + NO_3_^–^ production observed at 10 and 20^°^C, but exceeded soil nitrification rates observed at 30^°^C. When NH_4_^+^ was added to increase NH_3_-oxidation activity in this soil, there was no accumulation of NO_2_^–^ in the Coastal Plain soil above our limit of detection (2 μM NO_2_^–^), and under these conditions the MM models using kinetic parameters obtained for *Nitrospira* NOB in this study did not predict *Nitrospira* contributions to soil NO_2_^–^ oxidation (*R*^2^ = 0.09–0.32, Figure 3G). The greatest MM model-predicted response for *Nitrospira* was *N. moscoviensis*, but it would only account for ∼3% of the total soil NOB activity at its highest activity rates. In contrast, the MM model for the *Nitrobacter* NOB *N. vulgaris* predicted rates of NO_2_^–^ oxidation that met or exceeded the response observed by soil NOB, but the MM models did not predict that other *Nitrobacter* could contribute under these conditions with low concentrations of soil NO_2_^–^ (Figure 3H).

### Model Predictions Compared to Columbia Basin Soils

In Columbia Basin soils with no NH_4_^+^ addition, soil AOA had much higher rates of activity than that predicted by the MM model of *N. viennensis* (Figure 4A). In the absence of addition of NH_4_^+^ to soil, AOB had activity at 20, 30, and 40^°^C, but neither of the MM models using the kinetic parameters from *N. multiformis* and *N. europaea* had high enough substrate affinity to predict the oxidation of NH_3_ at such low concentrations (Supplementary Table 7 and Figure 4B). NO_2_^–^ did not accumulate in the Columbia Basin soil in the absence of the addition of NH_4_^+^; therefore, the rates of soil NOB activity matched that of NH_3_ oxidation. In the absence of accumulated NO_2_^–^, none of the *Nitrospira* NOB models predicted significant contributions under these low NO_2_^–^ conditions (Figures 4C,D).

When NH_4_^+^ was added to the Columbia Basin soil, there was no significant change in the rates of NH_3_ oxidation by soil AOA over the incubations without NH_4_^+^ addition at 10, 20, and 40^°^C, but the rates at 30^°^C declined ∼2-fold, suggesting evidence of substrate inhibition (Figure 4E). While the MM model for *N. viennensis* predicted high rates of AOA NH_3_ oxidation in excess of those observed in the Columbia Basin soil, the H model accurately predicted the soil AOA activity (*R*^2^ = 0.918, *p* = 0.000, Figure 4E). With the addition of NH_4_^+^ to Columbia Basin soils, there were significant increases in the rates of activity by soil AOB with maximum rates of NH_3_ oxidation at 30^°^C (Figure 4F). Both *N. europaea* and *N. multiformis* MM models describe the NH_3_ oxidation response to NH_4_^+^ addition at 10, 20, and 30^°^C, but overestimated the AOB response at 40^°^C; resulting in the models that poorly fit the soil AOB activity (Supplementary Table 7). Although the rates of NO_2_^–^ oxidation by soil NOB increased ∼2-fold in response to the higher rates of NH_3_ oxidation (Figures 4G,H), NO_2_^–^ accumulated in the Columbia Basin soil when NH_4_^+^ was added (2, 700, 1,900, and 400 μM NO_2_^–^ at 10, 20, 30, and 40^°^C, respectively), indicating that soil NOB did not have the capacity to immediately respond to higher rates of NH_3_ oxidation. However, the MM models of both *Nitrospira* and *Nitrobacter* NOB indicated a positive benefit in predicted rates of NO_2_^–^ oxidation from the accumulated NO_2_^–^, and only *N. moscoviensis* showed substrate-induced inhibition at 30 and 40^°^C (Figure 4G). The MM model for *Nitrospira* NOB predicted rates equal to or greater than soil NOB at 20 and 40^°^C, but could not match the rate of soil NOB at 10 or 30^°^C. The MM and H models with accumulated NO_2_^–^ for all three *Nitrobacter* NOB predicted the capacity to express a higher activity than the observed soil NOB activity but resulted in a significant fit of the soil data (*p* ≤ 0.004, Supplementary Table 7, and Figure 4H).

## Discussion

### The Effect of Temperature on Catalytic Efficiency

The *K*_*m*(app)_ of all three *Nitrobacter* species increased substantially with temperature at a much greater rate than *V*_*max*(app)_, resulting in a substantial decrease in catalytic efficiency at higher temperatures. In contrast, both *V*_*max*(app)_ and *K*_*m*(app)_ values of the *Nitrospira* species increased similarly with temperature, on a much smaller scale, and their overall catalytic efficiency generally did not change significantly. This difference between *Nitrobacter* and *Nitrospira* makes sense in view of the analysis of the phylogeny of NxrA suggesting that NO_2_^–^ oxidation has arisen independently multiple times (Lücker et al., 2010; Sorokin et al., 2012), resulting in different biochemical or functional characteristics. For example, the NXR complex is located in the periplasm space of *Nitrospira* sp. and in the cytoplasm space of *Nitrobacter* sp. (Daims et al., 2016), which may contribute to the observed differences in substrate affinity between the groups (*Nitrospira K*_*m*(app)_ = 9–24 μM, *Nitrobacter K_*m*_*_(app)_ = 49–544 μM; Nowka et al., 2015; Daims et al., 2016; Ushiki et al., 2017). Additionally, while there was a 50–70% decline in catalytic efficiency with increasing temperature for each of the three *Nitrobacter* evaluated in this study, there was more variation in the response of *Nitrospira*; ranging from a ∼50% decline in catalytic efficiency over 10 to 37^°^C by *Ns.* ND1, to no change in catalytic efficiency by *N. japonica NJ1*, and a uniform response in catalytic efficiency across temperature by *N. moscoviensis* except at 20^°^C where there was a 4-fold increase. The phylogeny of NxrA indicates that there is more diversity within *Nitrospira* than in *Nitrobacter* (Daims 2016 Trends), and all *Nitrobacter* evaluated in this study have very similar NxrA (92–98% identity) compared to lineages I and II *Nitrospira*. As for *N. japonica* and *N. moscoviensis*, their NxrA have only ∼87% sequence identity and are clearly separated by phylogenetic analysis even though both NOB are in lineage II; the differences in their adaptation to different growth temperatures (30 and 37^°^C, respectively) may have contributed to their phylogenetic separation and differences in physiological response. *N. japonica* NJ1 is similarly divergent compared to *Nitrospira* ND1 (lineage I, 88% identity; Figure 1B).

Although the limited number of NH_3_ oxidizer cultures utilized in this study prevents definitive conclusions on the response of catalytic efficiency to temperature change, the results agree with previous studies and are in sharp contrast to that of the NOB. Numerous studies have demonstrated higher rates of NH_3_ oxidation in natural environments as temperatures increase (Myers, 1975; Groeneweg et al., 1994; Avrahami et al., 2003; Tourna et al., 2008; Gubry-Rangin et al., 2017; Ouyang et al., 2017; Taylor et al., 2017; Duan et al., 2018, 2020; Mukhtar et al., 2019; Zheng et al., 2020; Bello et al., 2021), but there is a dearth of information on temperature-dependent changes of substrate affinity and catalytic efficiency. In this study, AOB showed 3–7-fold increases in *K*_*m*(app)_ with temperature (decrease in affinity), but greater increases in *V*_*max*(app)_ with temperature (8–16-fold), which resulted in 2.5–2.8-fold increases in catalytic efficiency. This agrees with previous results with *Nitrosomonas* sp. 4W30 that had a 1.9–2.7-fold increase in catalytic efficiency as temperatures increased from 5 to 20^°^C (Jones and Morita, 1985). In the case of the AOA *N. viennensis*, there was no significant change in *K*_*m*(app)_ but *V*_*max*(app)_ increased nearly two orders of magnitude resulting in a significant increase of catalytic efficiency as temperatures increased. This increase in catalytic efficiency with temperature by *N. viennensis* was similar to the response of the AOA “*Ca. N. oleophilus*” MY3 over 25–35^°^C where there was ∼2-fold increase in catalytic efficiency (Jung et al., 2022).

To date, it is unknown if temperature differentially affects NH_3_ and NO_2_^–^ oxidation in comammox *Nitrospira*. Previous evaluation of the kinetic parameters of NH_4_^+^ and NO_2_^–^ oxidation in comammox cultures of Nitrospira *inopinata* and “*Candidatus* Nitrospira kreftii” at their optimal growth temperatures (37^°^C and room temperature, respectively) found that the catalytic efficiency of NH_3_ oxidation was one to two orders of magnitude greater than that of NO_2_^–^ oxidation (Kits et al., 2017; Sakoula et al., 2020). The different catalytic efficiencies of NH_3_ oxidation (19.6) and NO_2_^–^ oxidation (0.05) of *N. inopinata* may have caused the accumulation of NO_2_^–^ until all NH_4_^+^ was consumed (Daims et al., 2015; Kits et al., 2017). In contrast, NO_2_^–^ did not accumulate in “*Candidatus* Nitrospira kreftii” cultures where there were smaller differences between the catalytic efficiency of NH_3_ (33–42) and NO_2_^–^ oxidation (4–8; van Kessel et al., 2015; Sakoula et al., 2020). Because the activity and catalytic efficiency of NH_3_ and NO_2_^–^ oxidizers affect N availability and productivity in soil, marine, and surface waters, and engineered systems, future studies are needed to investigate the coupling of nitrification, including comammox in response to temperature.

### A Few Observations on the Michaelis–Menten and Haldane Kinetic Parameters of NH_3_ and NO_2_^–^ Oxidation

Ammonia and NO_2_^–^ are poor energy sources to support an autolithotrophic lifestyle; therefore, it makes intuitive sense that the optimal growth temperature (*T*_*opt*_) of the NH_3_ oxidizers evaluated in this study was at, or near, the temperature where the response of MM kinetics resulted in near peak catalytic efficiency for NH_3_ oxidation (Figure 2). In contrast, the growth *T*_*opt*_ of the six NOB evaluated in this study did not occur at the temperatures where their catalytic efficiency of NO_2_^–^ oxidation was at its best. Under optimal growth conditions, it has been calculated that NOBs spend ∼26.7% of the energy generated from NO_2_^–^ oxidation in cellular homeostasis, while those demands rise greater than 3-fold under non-growth conditions (Vadivelu et al., 2006; Ni et al., 2008). Perhaps, NOBs prioritize cell maintenance over growth. *Nitrobacter* NOBs have multiple energy storage cell inclusions, such as poly-β-hydroxybutyrate, glycogen, and polyphosphate granules (Watson et al., 1989; Spieck and Bock, 2005), and the energy generated during the oxidation of NO_2_^–^ may be used to create or enrich these storage molecules until optimal growth conditions are met. We also observed that the H substrate inhibition of *N. viennensis, N. europaea, N. moscoviensis*, and *N. winogradskyi*, was temperature-sensitive (Supplementary Table 5), which may be another potential explanation for why *T*_*opt*_ for growth and the temperature for optimal catalytic efficiency are not aligned, for it has been long known that substrate inhibition can occur during the isolation and culture of nitrifiers (Both et al., 1992; Bartosch et al., 2002; Spieck et al., 2006; Spieck and Lipski, 2011; Tourna et al., 2011). The H inhibition constant *K*_*i*_ was the highest (lowest sensitivity) at the optimal growth temperature (30^°^C) of *N. winogradskyi* and *N. vulgaris*, suggesting that substrate sensitivity may determine the *T*_*opt*_ for growth rather than optimal catalytic efficiency. We also observed that *N. moscoviensis*, *N. japonica strain* NJ1, and *N. vulgaris* all had their highest sensitivity (*K*_*i*_ < 0.9 mM) to NO_2_^–^ at 10^°^C, which is interesting because Venterea et al. (2020) reported NO_2_^–^ accumulation in soil at temperatures of 5 and 10^°^C, whereas it was quickly consumed at 22 and 30^°^C. Intriguingly, we found that there was a trend for the AOA *N. viennensis* to be the most sensitive to NH_4_^+^ (*K*_*i*_ 0.3–0.5 mM) at its optimal growth temperature, which may help explain why it has been challenging to isolate AOA cultures.

Both *Nitrobacter* and *Nitrospira* have demonstrated the ability to utilize NO_3_^–^ as a terminal electron acceptor in the presence of organic molecules, using NXR in the reverse direction to reduce NO_3_^–^ to NO_2_^–^ (Aleem and Sewell, 1981; Tanaka et al., 1983; Sundermeyerklinger et al., 1984; Freitag et al., 1987; Meincke et al., 1992; Lücker et al., 2010); and in the case of *Nitrobacter*, NXR possessed a higher affinity for NO_3_^–^ (∼0.9 mM NO_3_^–^) than for NO_2_^–^ (0.54–3.6 mM NO_2_^–^). Intriguingly, the highest rates of ATP production were observed in whole-cell incubation of *N. winogradskyi* when both oxygen and NO_3_^–^ were provided as electron acceptors, suggesting the potential for NO_2_^–^ oxidation and NO_3_^–^ reduction to occur concurrently (Freitag and Bock, 1990). There are examples of temperature affecting the balance between the forward and reverse direction of enzymes in different ways. The equilibrium in favor of the forward reaction of glucose isomerase increased from 50 to 60^°^C (Dehkordi et al., 2009) while the affinity of the forward reaction of glycerol-2-phosphate dehydrogenase was higher at 5^°^C compared to 21^°^C or 37^°^C (Ruberto et al., 2019). It is unknown how temperature will affect the balance between the forward and reverse actions of NXR, or if NXR of *Nitrobacter* and *Nitrospira* will respond in different ways.

### Protein Structure or Membrane Differences Between Groups

Bacterial and archaeal AMOs are integral membrane proteins (Norton et al., 2002; Urakawa et al., 2011), NXRs of *Nitrobacter* are membrane-bound proteins (Sundermeyerklinger et al., 1984; Meincke et al., 1992), and NXRs of *Nitrospira* are soluble proteins anchored to membranes (Mundinger et al., 2019); and it may be worth considering differences in membrane lipid composition between lineages to explain dissimilar temperature responses. This is based on (i) membrane dynamics and fluidity change in response to temperature, and the degree to which they change depends on lipid composition and lipid phase transition temperature (Mansilla et al., 2004), (ii) membrane protein activity and stability can be dependent on lipid composition and dynamics (Hong and Tamm, 2004; Reading et al., 2017; Sanders et al., 2018), (iii) lipid composition of *Nitrobacter* and *Nitrospira* is quite different (Lipski et al., 2001), indicating that their lipid membranes will respond differently to temperature, and (iv) AOA and other Thaumarchaeota have a high proportion of crenarchaeol, a core lipid that is unique in structure and potentially function from those of bacteria (Pitcher et al., 2010; Elling et al., 2014). It is well known that the archaeal and bacterial membrane lipid composition is dependent on growth temperature and is often used as a proxy for past temperatures (Elling et al., 2014; De Jonge et al., 2019), and that both bacteria and archaea actively modify lipid membrane composition in response to transient changes in temperature (Balogh et al., 2013; Siliakus et al., 2017). The cyclization of thaumarchaeal glycerol dialkyl glycerol tetraethers (GDGTs) membrane-spanning lipids is strongly correlated with sea surface temperature (Elling et al., 2014; Qin et al., 2015), and the degree of methylization and cyclization of branched bacterial glycerol dialkyl glycerol tetraethers (GDGTs) that are abundant in terrestrial and aquatic systems varies with the mean annual temperature (De Jonge et al., 2019). While NH_3_ and NO_2_^–^ oxidizers may have the ability to modify the lipid composition of cellular and periplasmic membranes in response to transient temperature changes, the incubations in this study were likely not long enough for such changes to occur. However, the cultures in this study grown under optimal conditions would differ in lipid composition in ways that could influence lipid dynamics, and may be as important as variation in protein structure in governing the temperature response of individual nitrifying organisms.

### Implications of Changes in Catalytic Efficiency in Natural Environments

Based on these data, increasing temperature should result in an increase in the catalytic efficiency of NH_3_ oxidation, while *Nitrospira* NOBs are not predicted to increase catalytic efficiency with temperature, and the catalytic efficiency of *Nitrobacter* NOB declines significantly. This differential change in catalytic efficiency may help explain the accumulation of NO_2_^–^ previously observed in other studies. For example, increasing temperature decoupled NO_2_^–^ oxidation from NH_3_ oxidation in coastal waters (Heiss and Fulweiler, 2016; Schaefert and Hollibaugh, 2017), and in multiple soil types (Giguere et al., 2017; Taylor et al., 2019; Duan et al., 2020; Waggoner et al., 2021). We have previously observed that the accumulation of NO_2_^–^ was required to stimulate the rates of NO_2_^–^ oxidation to match those of NH_3_ oxidation in a range of soils (Giguere et al., 2018; Taylor et al., 2019); but accumulated NO_2_^–^ also has the potential to inhibit NOB activity (Park and Bae, 2009; Zheng et al., 2021; Supplementary Table 5). Rates of NO_2_^–^ oxidation were stimulated ∼2-fold across all temperatures when NO_2_^–^ accumulated in NH_4_^+^-amended Columbia Basin soil incubations (Figure 3); however, these concentrations were greater than the temperature-dependent *K*_*i*_ of four of the NOB cultures evaluated in this study, and our modeling suggests temperature-dependent H inhibition, particularly of *Nitrospira* NOB.

Changes in enzyme catalytic efficiency with temperature are not specific to nitrification, and previous studies of some enzyme activities linked to processing of organic C, N, and P (cellobiohydrolase, β-glucosidase and xylanase, phosphatase, leucine-aminopeptidase, and tyrosine-aminopeptidase) in soil have observed changes in catalytic efficiency in response to temperature. Catalytic efficiency was positively correlated with temperature at sites ranging from boreal to tropical forests (German et al., 2012) and in long-term N addition plots (Stone et al., 2012). In other studies, catalytic efficiency increased with temperature or did not change depending on the enzyme evaluated (Blagodatskaya et al., 2016; Razavi et al., 2016). Curiously, none of these studies observed pronounced declines in catalytic efficiency as temperature increased as was observed in this study with *Nitrobacter* NOB.

## Data Availability Statement

The original contributions presented in the study are included in the article/Supplementary Material, further inquiries can be directed to the corresponding author.

## Author Contributions

AT was responsible for experimental design, data analysis, and writing of the manuscript. BM was responsible for execution of experiments, phylogenetic analysis, and writing of the manuscript. Both authors contributed to the article and approved the submitted version.

## Conflict of Interest

The authors declare that the research was conducted in the absence of any commercial or financial relationships that could be construed as a potential conflict of interest.

## Publisher’s Note

All claims expressed in this article are solely those of the authors and do not necessarily represent those of their affiliated organizations, or those of the publisher, the editors and the reviewers. Any product that may be evaluated in this article, or claim that may be made by its manufacturer, is not guaranteed or endorsed by the publisher.

## Footnotes

1
https://soilseries.sc.egov.usda.gov

2
https://www.ncbi.nlm.nih.gov/

3
http://www.genome.jp/kegg/

4
http://blast.ncbi.nlm.nih.gov/Blast.cgi

5
http://tree.bio.ed.ac.uk/software/figtree/
