# Supplementary material for: Differential Responses of the Catalytic Efficiency of Ammonia and Nitrite Oxidation to Changes in Temperature
Source: Front Microbiol. 2022 May 10;13:817986. doi: 10.3389/fmicb.2022.817986 (PMC9127996; doi:10.3389/fmicb.2022.817986)
Supplement: Supplementary file 1 [file Data_Sheet_1.zip › Supplemental Dataset S1.DOCX]

#Genus_species_protein_genbankID

>Neuro_AmoA_CAD84855.1

MSIFRTEEILKAAKMPPEAVHMSRLIDAVYFPILIILLVGTYHMHFMLLAGDWDFWMDWK
DRQWWPVVTPIVGITYCSAIMYYLWVNYRQPFGATLCVVCLLIGEWLTRYWGFYWWSHYP
INFVTPGIMLPGALMLDFTLYLTRNWLVTALVGGGFFGLLFYPGNWPIFGPTHLPIVVEG
TLLSMADYMGHLYVRTGTPEYVRHIEQGSLRTFGGHTTVIAAFFSAFVSMLMFTVWWYLG
KVYCTAFFYVKGKRGRIVHRNDVTAFGEEGFPEGIK

>Neutropha_AmoA_ABI60535

MSIFRTEEILKAAKMPPEAVHMSRLIDAVYFPILVVLLVGTYHMHFMLLAGDWDFWMDWK
DRQWWPVVTPIVGITYCSAIMYYLWVNYRQPFGATLCVVCLLIGEWLTRYWGFYWWSHYP
LNFVTPGIMLPGALMLDFTMYLTRNWLVTALVGGGFFGLMFYPGNWPIFGPTHLPIVVEG
TLLSMADYMGHLYVRTGTPEYVRHIEQGSLRTFGGHTTVIAAFFAAFVSMLMFAVWWYLG
KVYCTAFFYVKGKRGRIVQRNDVTAFGEEGFPEGIK

>Nmult_AmoA_ABB74106.1

MSRTDEILKAAKMPPEAVKMSRMIDVIYFPILCILLVGTYHMHFMLLAGDWDFWLDWKDR
QWWPVVTPIVGITYCAAIMYYLWVNYRLPFGATLCIVCLLVGEWLTRFWGFYWWSHYPMN
FVFPSTMIPGALVMDTVLLLTRNWMITALVGGGAFGLLFYPGNWTIFGPTHLPLVAEGVL
LSVADYTGFLYVRTGTPEYVRLIEQGSLRTFGGHTTVIASFFSAFVSMLMFTVWWYFGKV
YCTAFYYVKGARGRVSMKNDVTAFGEEGFAEGIK

>Nviennensis_AmoA_AIC17003.1

MVWLRRTTHYIFIVVVAVNSTLLTINAGDYIFYTDWMWTSFVVFSVSQSTMLAVGAVYYM
LFTGVPGTATYYATIMTIYTWVAKGAWFALGYPYDFIVVPVWIPSAMLLDLTYWATRRNK
HAVIIIGGTLVGLSFPLFNMVNLLLVRDPLEVAFKYPRPTLPAYMTPIEPQVGKFYNSPV
ALGSGASAVLTVPMTALGAKLNTWTYRWMAAWSKWD

>Nbrien_AmoA_WP_025042266.1

MSRTDEILKAAKMPPEAVKMSRMIDAVYFPILCILLVGTYHMHFMLLAGDWDFWLDWKDRQWWPVVTPIVGITYCAAIMY

YLWVNYRLPFGATLCVVCLLTGEWLTRYWGFYWWSHYPISFVFPSTMIPGALVMDTVMLLTRNWMITALVGGGAFGLLFY

PGNWPIFGPTHLPLVAEGVLLSVADYTGFLYVRTGTPEYVRNIEQGSLRTFGGHTTVIASFFAAFVSMLMFCLWWYFGKL

YCTAFFYVKGARGRVTMKNDVTAFGEEGFPEGIK

>Nlacus_AmoA_ARO87402.1

MSRTDEILKAAKMPPEAVKMSRMIDAVYFPILCILLVGTYHMHFMLLAGDWDFWLDWKDR
QWWPVVTPIVGITYCATIMYYLWVNYRLPFGATLCIVCLLTGEWLTRFWGFYWWSHYPIN
FVLPSTMIPGALIMDTVLLLTRNWMITALVGGGAFGLLFYPGNWPIFGPTHLPLVAEGVL
LSLADYTGFLYVRTGTPEYVRLIEQGSLRTFGGHTTVIAAFFSAFVSMLMFCVWWYFGKL
YCTAFYYVKGPRGRVTMKNDVTAYGEEGFPEGIK

>Ngargensis_AmoA_AFU59457

MFIVVVAVNSTLLTINAGDYIFYTDWMWTSFVVFSISQSTMLVVGAIYYMLFTGVPGTAT
YYATIMTIYTWVAKGAWFALGYPYDFIVVPVWIPSAMLLDLTYWATRRNKHAAIIIGGTL
VGLSFPLFNMVNLLLVRDPLEVAFKYPRPTLPAYMTPIEPQVGKFYNSPVALGSGAGAVL
SVPIAALGAKLNTWTYRWMAAWSKWD

>Noleophilus_AmoA_ALI37465

MVWLRRTTHYLFIVVVAVNSTLLTINAGDYIFYTDWAWTSFVVFSISQSTMLVVGAIYYM
LFTGVPGTATYYATIMTIYTWVAKGAWFALGYPYDFVVVPVWIPSAMLLDLAYWATRRNK
HAAILIGGVLVGMSLPLFNMINLLLVADPLEMAFKYPRPTLPPYMTPIEPQVGKFYNSPV
ALGAGAGAVLCVPIAALGAKLNTWTYRWMAAWSKWD

>Ninopinata_AmoA_CUQ66826

MFRTDEIIKAAKLPPEGVAMSRHIDYIYFIPILFVTIIGTFHMHTALLCGDWDFWLDWKD
RQWWPIVTPITTITFCAALQYYNWVNYRQPFGATITILALGAGKWVAVYTSWWWWSNYPP
NFVMPATLLPSALVLDFTLLLTRNWTLTAVIGAWMYAILFYPSNWPIFAYSHTPLVVDGT
LLSWADYMGFMYVRTGTPEYIRMIEVGSLRTFGGHSTMISSFFAAFASSLMYILWWQFGK
FFCTSYFYFTDDKKRTTKVYDVFAYATLAHADKAKLSGGKA

>Nnitrosa_AmoA_WP_090742150

mfrtdeiika sklppegvam srhidhiyfi pilfitivgt fhmhtallcg dwdfwldwkd

rqwwpivtpi ttitfcaalq yynwvnyrqp fgatitilal gfgkwiavyt swwwwsnypp

nfvmpatllp salvlditll ltrnwtltav igawmyailf ypsnwpifgy shtpivvdgs

llswadymgf myvrtgtpey irmievgslr tfgghstmis affsafassl vyvlwwqfgk

ffctsyfyft ddrqrtvkvy dvfayatlah gdkakvggka

>Nkreftii_AmoA_QPD05396

mfrtdeiika aklppegvam srhidyiyfi pilfvtivgt fhmhfdllag dwdfwidwkd

rqwwpivtpi taitfcaalq yynwvnyrqp fgaticilal lagkwvtiwa awwwwsnypv

nfvmpstllp saivldcill ltrnwtltav igawlfailf yptnwaifay shtplvvdgt

llswadymgf ayirtgtpey irmievgslr tfgghstmis sffaafassl myilwwqfgk

ffctsyfylt ddrqkttkvy dvfayatlah gdkaklsggk a

>Mheyeri_PmoA_QGM45338

MSTSKSGGAVGPFHSVAEAAGCVQTTDWLLLTLLFFAVLGGYHVHFMLTAGDWDFWVDWK
DRRMWPTVVPILGVTFAAASQAFFWVNFRLPFGATFAVLGLLIGEWINRYVNFWGWTYFP
ISLVFPSALMVPAIWLDVILLLSGSYVITAIVGSLGWGLLFYPNNWPAIAAFHQATEQHG
QLMTLADLIGFHFVRTSMPEYIRMVERGTLRTFGKDVVPVAAFFSGFVSMMVYFLWWFMG
RWYSTTAKIEKI

>Noceani_AmoA_WP_011330982.1

msaltsavrt peeaakvsrt ldfialgaff millashhvh vmllmgdwdf wvdwkdrrfw

vtvvpivsva ypaaaqaffw ekfrlpfgat lvtlgvlage wanryfnfvg ftyfpinfvw

ptillpmalf ldamlaisks ygltavvggl mygllmypan wpllsafhvp aeyngvvmsl

adimgyqyvr tgtpeyirmi ekgtlrtfgk dvvpvsaffs gfvamvmyfv whfvgrwfsk

dyhidqv

>Nmobilis_AmoA_SCZ85183

mnefllaliy ivpllmvltl ltkgeglltg vfrtneilka akmppeaihm srlidavyfp

ilivllvgty hmhfmllagd wdfwldwkdr qwwpvltpiv gitycsaimy ylwvnyrqpf

gatlcvicll lgewltrywg fywwshypin fvtpgimlpg almldltlyl trnflitall

ggaffgllfy pgnwtifgpt hlpivveghl lsmadymghl yirtgtpeyt rliekgslrt

fgghttviaa ffasfvsmlv flvwwylgkv yctaffyvkg krgrivhred vtafgeegfp

erik
