# Supplementary material for: Differential Responses of the Catalytic Efficiency of Ammonia and Nitrite Oxidation to Changes in Temperature
Source: Front Microbiol. 2022 May 10;13:817986. doi: 10.3389/fmicb.2022.817986 (PMC9127996; doi:10.3389/fmicb.2022.817986)
Supplement: Supplementary file 1 [file Data_Sheet_1.zip › Supplemental Dataset S2.DOCX]

#Genus_species_protein_genbankID

>Nmoscoviensis_NxrA_ALA56694

MFLSRRQFLKVSAGTVAAVAVADKVLALTALQPVIEVGNPLGDYPDRSWERVYHDQYRYD
SSFTWCCSPNDTHACRIRAFVRNGVVMRVEQNYDHQTYEDLYGNRGTFAHNPRMCLKGFT
FHRRVYGPYRLKGPLMRKGWKQWMDDGSPELTPETKRKYKFDSRFLDDMLRVSWDTAFTY
AAKAMIIVATRYSGEAGARRLREQGYAPEMIEMMKGAGTRCFKHRAGMPVLGILGKMGNT
RMNGGINALLDTWIRKVSPDQAQGGRYWSNYTWHGDQNPAHPWWSGAQGSDIDLSDMRFS
KLNTSWGKNFVENKMPEAHWKLECIERGARVVVITPEYNPTAYRADYWMPLRPESDGSLF
LGAMKIIVDENMHDIDFLKSFTDAPILVRTDTLQYLDPRDVVADYKFPDFSKSYSGRIQS
LKPEQIERLGGMMVWDLNKKQAVPLHREQVGWHYVNSGIDAALTGTYRVKLLNGREIDAM
PVWQMYLVHFQDYDLDTVHQICRTPKDLIVRWARDSGTIKPAAIHNGEGTCHYFHQTINA
RGAAMVLIITGNVGKFGTGQHTWAGNYKAGTWTATPWSGAGLAVHTGEDPFNITTDPNAH
GKEIKTKSYYYGEEVGYWNHGDTALIVNTPKYGRKVFTGKTHMPTPSKLRWVTNVNVLNN
AKHHYDMVRNVDPNIETLITQDIEMTSDVNHNDIAFACNSWMEFTYPEMTVTVSNPWVQI
WKGGIRPLYDTRNDLDTFAGTAAKLSEMTGDKRMKDYFAMVYLNRVDVYAQRMLDASSTF
YGYSADTMLKSEKGWMVMVRTYPRHPFWEETNESKPMWTRSGRYENYRIEPEAIEYGENF
ISHREGPEATPYLPNAIFTTNPYCRPDDYGIPITAQHHDDKTVRNIKLSWHEIKRHSNPL
WEKGYQFYCVTPKTRHRVHSQWSVNDWVQIYESNFGDPYRMDKRTPGVGEHQLHINPQAA
KDRGINDGDYVYVDGNPVDRPYRGWKPSDPYYKVARLMIRAKYNPSYPYHVTMAKHAPYV
STAKSVKGHETRPDGRAIAVDTGYQSNFRYGAQQSFTRNWLMPMHQTDSLPGKHAIAWKF
KWGYQVDHHAINTVPKECLIRITKAEDGGIGARGPWEPVRTGFTPGQENEFMIKWLKGEH
IKIKV

>Njaponica_NxrA_SLM46989

MVWSRRQFLKISAGTVAAVAVADKVLALTALQPVIEVGNPLGDYPDRSWERVYHDQYRYD
SSFTWVCSPNDTHACRIRAFVRNGVVMRVEQNYDHQTYEDLYGNRGTFAHNPRMCLKGFT
FHRRVYGPYRLKGPLMRKGWKQWMDDGSPELTPETKRKYKFDSRFLDDMIRVSWDTAFTY
AGKGLIVIGTRYSGEAGARRLREQGYAPEMIEMMKGAGVRCFKHRAGMPILGLLGKHGNT
RFNNSVLPLVDAWIRKVGPDQAQGGRYWNNYTWHGDQDPSQPWWNGTQNCDTDLSDMRFS
KLNTSWGKNFTENKMPEAHWKLESIERGGRIVVITPEYNPTAYRADYWIPVRPESDGALF
LGASRIIVEENLHDIDFLKQFTDMPLLVRTDTLQYLDPRDVVKDYALPDFSHSYSGRIQG
LKPEYIQRLGGMMVWDLNKKQAVPLHREQVGVHYQETGVDAALTGTYRVKMLNGREVDVA
PIYQLYMVHFQDYDLDTVHQINRSPKDLIVRWARDCGTVKPAAIHNGEGVCHYFHMTSMG
RAAALVMILTGNVGKFGTGCHTWSGNYKVGIWNAAPWSGAGAGVHLSEDPWHTNLDPNAH
GKEIHYKTYYYGEEPGYWNHGDTALIVNTPKYGRKVFTGKTHMPSPSKVRWVVNVNVLNN
SKHHYDMVRNVDPNIEMIITQDIEMTSDVNHADIAFACNSWMEFTYPEMTATVSNPWIQV
WKGGIRPLYDTRNDLDTFAGVAAKLSEMTGDTKIRDVFHFVYLNRVDVYAQRVFDASSTL
YGYSADVMLKSEKGWMVMVRTYPRHPLWEEVNESKPQWTRSGRLETYRVEPEAIEYGENF
IVHREGPEATPYLPNAIFTTNPYVRPDDYGIPITAQHHDDKTVRNIKLPWGEIKQHANPL
WEKGYQFYCVTPKTRHRVHSQWSVNDWVQIYESNFGDPYRMDKRTPGVGEHQLHINPQAA
KDRGINDGDYVYVDGNPVDRPYRGWKPSDPYYKVARLMIRAKYNPAYPYHVTMAKHAPYV
STAKSVKGHETRPDGRAIAVDTGYQSNFRYGAQQSFTRSWLMPMHQTDSLPGKHANGLKW
KWGFEIDHHAVNTVPKECLIRITKAEDGGIGARGPWEPVRTGFTPGQENEFMIKWLKGEH
IKIKV

>NND1_NxrA_WP_080880476

mmqlsrrqfl kvsagtvava avadkalalt alqpvvevdn plgeypdrsw ervyhdqyry

dssftwccsp ndthacrira fvrngvvmrv eqnydhqtye dlygnrgtfa hnprmclkgf

tfhrrvygpy rlkgplmrkg wkqwmddgsp eltsdakrky kfdsrflddm vrvswdtaft

yvakglivig trysgeagar rlreqgyape miemmkgagv rtfkhragmp ilgmmgkhan

trfnncvlpl ldswirkvnp dqaqggrywn nytwhgdqdp sqpwwngtqn cdvdlsdmrf

tklntswgkn fvenkmpeah wklesmerga rlvvitpeyn ptasradywi pvrpetdgal

flgaskiild enyqdiefik gftdmpllvr tdtlqyldph evlrdyqvpd ftksysgrvq

gltqdqvqrl ggmmvwdlak gkavplhreq vglhlaqsgi dpaltgtyrv kllngrevdv

mpiyqlytih lqdydldtvh qvnrspkdli vrwardcgtv kpaaihngeg vchyfhmtsm

graaalvmml tgnigkfgtg chtwsgnykv giwqaapwsg agasvylged pwnlnlrddv

hgkeikyrky yygeepgywn hgdnalivnt pkygrkvftg kthmpspskv rwvvnvniln

nakhhydmvk nvdpniemli tqdiemtsdv nhadvafavn swmeftypem tatvsnpwvq

iwkggirply dtrndldsfa gvaaklkeit geqrmadtyk fvyhnrvdiy vqrildasst

ffgysadvml ksekgwmvmc rtyprhplwe etneskphwt rsgrletyri epeaieygen

fishregpec tpympnaimt tnpyvrpedy gipvtaqhhd dktvrniklp wseikqhanp

lwekgyqfyc vtpktrhrvh sqwsvndwvq iyesnfgdpy rmdkrtpgvg ehqihinpqa

akdrgindgd ycyvdgnpvd rpyrgwkpsd pfykvarlmi rakynpsypy hvtmakhapy

vstaksvkgh etrpdgraia vdtgyqsnfr ygaqqsftrs wlmpmhqtds lpgkqanalk

fkwgfeidhh avntvpkecl iritkaedgg igargpwepv rtgftpgqen efmikwlkge

hikikv

>Ndefluvii_NxrA_CBK42928

MMQLSRRQFLKVSAGTVAVAAVADKALALTALQPVVEVNNPLGEYPDRSWERVYHDQYRY
DSSFTWVCSPNDTHACRIRAFVRNGVVMRVEQNYDHQTYEDLYGNRGTFAHNPRMCLKGF
TFHRRVYGPYRLKGPLMRKGWKQWMDDGSPELTSDVKRKYKFDSRFLDDMVRVSWDTAFT
YVAKGLIVIGTRYSGEAGARRLREQGYAPEMIEMMKGAGVRTFKHRAGMPILGMMGKHAN
TRFNNCVLPLLDSWIRKVNPDQAQGGRYWNNYTWHGDQDPSQPWWNGTQNCDVDLSDMRF
TKLNTSWGKNFVENKMPEAHWKLESMERGARLVIITPEYNPTASRADYWIPVRPETDGAL
FLGASKIILDENYQDIDFIKGFTDMPLLVRTDTLQYLDPHEVLKDYQVPDFTKSYSGRVQ
GLSQDQVQRLGGMMVWDLAKGKAVPLHREQVGVHLAQSGIDPALTGTYRIKLLNGREVDV
MPIYQLYTIHLQDYDLDTVHQVNRAPKDLIVRWARDCGTVKPAAIHNGEGVCHYFHMTSM
GRAAALVMMLTGNIGKFGTGCHTWSGNYKVGIWQAAPWSGAGASVYLGEDPWNLNLRDDV
HGKEIKYRKYYYGEEPGYWNHGDNALIVNTPKYGRKVFTGKTHMPSPSKVRWVVNVNILN
NAKHHYDMVKNVDPNIEMLVTQDIEMTSDVNHADVAFAVNSWMEFTYPEMTATVSNPWVQ
IWKGGIRPLYDTRNDLDSFAGVAAKLKEMTGEQRMADTYKFVYHNRVDIYVQRILDASTT
FFGYSADVMLKSEKGWMVMCRTYPRHPLWEETNESKPHWTRSGRLETYRIEPEAIEYGEN
FISHREGPECTPYMPNAIMTTNPYVRPEDYGIPVTAQHHDDKTVRNIKLPWSEIKQHPNP
LWEKGYQFYCVTPKTRHRVHSQWSVNDWVQIYESNFGDPYRMDKRTPGVGEHQIHINPQA
AKDRGINDGDYCYVDGNPVDRPYRGWKPSDPFYKVARLMIRAKYNPSYPYHVTMAKHAPY
VSTAKSVKGHETRPDGRAIAVDTGYQSNFRYGAQQSFTRSWLMPMHQTDSLPGKQANALK
FKWGFEIDHHAVNTVPKECLIRITKAEDGGIGARGPWEPVRTGFTPGQENEFMVKWLKGE
HIKIKV

>Ninopinata_NxrA_CUQ65854

mflsrrqflk vsvgtvaava vadkvlalta lqpvievgnp lgeypdrswe rvyhdqyryd

ssftwvcspn dthacrvraf vrngvvmrve qnydhqtyed lygnrgtfah nprmclkgft

fhrrvygpyr lkgplmrkgw kqwmddgape ltpetkrkyk fdsrflddml rvswdtafty

aakamiiiat rysgeagarr lreqgyapem iemmkgagtr cfkhragmpv lgiigkmgnt

rmngginall dtwirkvgpd qaqggrywsn ytwhgdqnpa hpwwsgvqgs dvdlsdmrfs

klntswgknf venkmpeahw kleciergar vvvitpeynp tayradywmp lrpqsdgalf

lgamkiivde nmhdvdflks ftdapilvrt dtlqyldprd vipdykfpdf sksysgriqs

lkpeqiqrlg gmmvwdlnkk qavplhreqv gwhyvnsgid aaltgtyrvk llngreidam

piwqmymvhf qdydldtvhq itrtpkdliv rwardsgtik paaihngegt chyfhqtina

rgaamvliit gnvgkfgtgq htwagnykag awtatpwsga glsvhtgedp fnitldpnah

gkeihtrsyy ygeevgywnh gdtalivntp kygrkvftgk thmptpskfr wvtnvnvvnn

akhhydmvkn vdpnieclit qdiemtsdin hadiafacns wmeftypemt itvsnpwvqi

wkggirplyd trndldtfag vaaklsdmtg dkrmkdyfam vyanrvdvya qrmldasstf

ygysadvmlk sekgwmvmvr typrhpfwee tneskpmwtr sgryenyrpe aeaieygenf

ishregpeat pylpnaiftt npyvrpddyg ipitaqhhdd ktvrniklsw deikrhsnpl

wekgyqfycv tpktrhrvhs qwsvndwvqi yesnfgdpyr mdkrtpgvge hqlhinpqaa

kdrgindgdy vyvdgnpvdr pyrgwkpsdp yykvarlmir akynpaypyh vtmakhapfv

atpksvkghe trpdgraiai dtgyqsnfry gcqqsftrnw lmpmhqtdsl pgkhaiawkf

kwgyqvdhha intvpkecli ritkaedggi gargpwepvr tgftpgqene fmikwlkgeh

ikikv

>Nnitrosa_NxrA_CUS33249.1

mflsrrqflk vsvgtvaaaa vadkvlalta lqpvievgnp lgdypdrswe rvyhdqyryd

ssftwvcspn dthacrvraf vrngvvmrve qnydhqtyed lygnrgtfah nprmclkgft

fhrrvygpyr lkgplmrkgw kqwmddgspe ltpdtkrkyk fdsrflddml rvswdtafty

aakamiiiat rysgeagarr lreqgyapem iemmkgagtr cfkhragmpv lgiigkmgnt

rmngginall dtwirkvspe qaqggrywsn ytwhgdqnps qpfwsgvqgs didladmrfs

klntswgknf venkmpeahw kleciergar vvvitpeynp tayradywmp lrpqsdgalf

mgamkiiide nmhdvdflkq ftdapilvrt dtlqyldprd vvadykfpdf sksysgriqa

lkpqdverlg gmmvwdlnkk qavplhreqv gwhyinsgid aalngtyrvk llngrevdam

pvwqmylvhf qdydldtthq icrtpkdliv rwardsgtik paaihngegv thyfhmtpng

raaamvliit gnvgkfgtgq htwagnykag cwtatpwsga glsvhtgedp fnitldpnah

gkeiktksyy ygeevgywnh gdtalivntp kygrkvftgk thmptpskfr wvvnvnvlnn

akhhydmvrn vdpnietlit qdiemtsdvn hndiafacns wmeftypemt itvsnpwvqi

wkggirplyd trndldtfag vaaklsemtg dkrmkdyfam vyanrvdvya qrmldasstf

ygysadvmlk sekgwmvmvr typrhpfwee tneskpmwtr sgryenyrie peaieygenf

ishregpeat pylpnaiftt npycrpddyg ipitaqhhdd ktvrniklsw heivrhsnpl

wekgyqfycv tpktrhrvhs qwsvndwvqi yesnfgdpyr mdkrtpgvge hqihinpqaa

kdrgindgdy vyvdgnpvdr pyrgwkpsdp yykvarlmir akynpaypyh vtmakhapyv

stpksvkghe trpdgraiai dtgyqsnfry gaqqsftrnw lmpmhqtdsl pgkhaiawkf

kwgyqvdhha intvpkecli ritkaedggi gargpwepvr tgftpgqene fmikwlkgeh

ikikv

>Nkreftii_NxrA_QPD06216

mflsrrqflk vsagtvaaaa vadnvlalta lqpvievgnp lgdypdrswe rvyhdqyryd

ssftwvcspn dthacrvraf vrngvvmrve qnydhqtyed lygnrgtfah nprmclkgft

fhrrvygpyr lkgplmrkgw kqwmddgspe ltpetkrkyk fdsrflddml rvswdtafty

aakamiviat rysgeagarr lreqgyapem iemmkgagtr cfkhragmpv lgivgkmgnt

rmngginall dtwirkvgpd qaqggrywsn ytwhgdqnps qpfwsgvqgs didlsdmrfs

klntswgknf venkmpeahw kleciergar vvvitpeynp tayradywmp lrpesdgalf

lgamkiiide nmhdtdfmkq ftdapilvrt dtlqyldprd vvadyqfpdf sksysgriqs

lkpeqiqrlg gmmiwdltkk qavplhreqv gwhyinsgid aaltgtyrvk llngreidam

pvwqmylvhf qdydldtvhq icrtpkdliv rwardsgtik paaihngegv chyfhmtpng

raaamvliit gnvgkfgtgq htwagnykag twtatpwsga glsvhtgedp fhitldpnah

gkeiktksyy ygeevgywnh gdtalivntp kygrkvftgk thmptpskfr wvvnvnvlnn

akhhydmvrn vdpnieclit qdiemtsdvn hndiafacns wmeftypemt vtvsnpwvqi

wkggirplyd trndldtfag vaaklsdmtg dkrmrdyfam vyanrvdvya qrmldasstf

ygysadvmlk sekgwmvmvr typrhpfwee tneskpmwtr sgryenyrie pesieygenf

ishregpeat pylpnaiftt npyvrpddyg ipitaqhhdd ktvrniklsw heimrhanpl

wekgyqfycv tpktrhrvhs qwsvndwvqi yesnfgdpyr mdkrtpgvge hqihinpqaa

kdrgindgdy vyvdgnpvdr pyrgwkpsdp yykvarlmir akynpsypyh vtmakhapyv

staksvkghe trpdgraiai dtgyqsnfry gaqqsftrnw lmpihqtdsl pgkhaiawkf

kwgyqvdhha intvpkecli ritkaedggi gargpwepvr tgftpgqene fmikwlkgeh

ikikv

>Nwinogradskyi_NxrA_ABA04039

MSWILDLVNPRERKWEEFYRNRWSHDNVFRSTHGVNCTGGCSWAIYVKDGIITWEMQQTD
YPLLERSLPPYEPRGCQRGISASWYVYSPIRVKYPYVRGPLYDLWKEAKASHPDPVQAWA
SLVGDEQKRSRIQKARGKGGFRRAKWEELVELIAAAALYTARKYGPDRVMGFSPIPAMSM
LSYAAGSRFLQLFGGVNMSFYDWYADLPTSFPEIWGDQTDVCESADWYNSKFIVSMAANM
NMTRTPDVHFISEARTEGTKFVVLSPDFSQIAKYCDEWIPIQAGQDTALWMAANHVILKE
YYIDRQVPYFIDYVKRYTDLPFLVELEPNGNTYKTGRLLRSNRVARYKDVENGEWKMLVL
DTATGEPRAFKGQVGDRWGSTHGKWNLSAEDTLDNSPIDPVLSFIDQSDGVVQVGFDDFV
NGSVVSRGVPVKRIATDKGEVLVTTGFDIMMSQFGHSRGLEGSFATSYDDEDAPYTPAWQ
ERHTGIGRETAIRFAREFATNAELTNGKSMVIVGASANHWYYNNLCYRSATVALILCGCC
GVNGGGINHYVGQEKLAPVAPWNTIAMALDWTKPPRVVQSSTWHYAHSCQWRYEQEFTEY
GLTAPNPRWAKGHAIDLEAKSVRCGWMPFTPHFNRNPIELAAEAERAGAKSTADIVTHVV
DQVASKKVNFAIDDPDAEESWPRMWFIWRGNAIQSSAKGHEFFLRHYLGAHDNSIAEDRA
KGKTQRVKYRDTAPRGKYDLVVDLNFRMNTTSLYSDIVLPTAFWYEKNDLNTTDLHSFLH
VLGQAVPPVWESKTDWEIFKLISKKVSELSPLAFSKPVRDVVVQPLMHDTPDELAQPEIL
DWAEGECKPVPGKSFPHVRVVERDYANLYNKFISFGPKAREDGVSAVGVNIPIKKQYDQM
LDNPIMPMPDARHMRCVEWGGKRYPSLEDVLDACNTLLMCAPEANGEVCYQGFHNEEHHV
GLPLVDIAEPTRGVSSTFYDLTRQPRRILTSPCWTGLTNDGRAYSAWCMNVERLVPWRTL
TGRQSLYLDHQWYLDFGEHIPTYKPRLNPRKTGDIVKSRVDDRSLVLNYITPHGKWNIHS
TYKDNHRMLMLSRGMDPVWINDRDAEKVGIEDNDWVEVYNDNGVVVTRANVSRRIQPGTC
MYYHAVERTVYIPKSQERKWRGGGHNSLTRTRINPLFLAGGYAQFTYGFNYWGPTGIFTR
DTHVVVRKMEKLEW

>Nhamburgensis_NxrA_ABE63726

MTTGLAAGQRTEETQMSWILDLVNPRERKWEEFYRNRWSHDNVFRSTHGVNCTGGCSWAI
YVKDGIITWEMQQTDYPLLERSLPPYEPRGCQRGISASWYVYSPIRIKYPYVRGPLLDMW
REAKASSADPVQAWGALVGDEQKRARMQKARGKGGYRRAKWEELVELIAAASLHTARKHG
PDRIMGFSPIPAMSMLSFAAGTRFLSLMGGSLLSFYDWYADLPTSFPEIWGDQTDVCESA
DWYNSKFIVSMASNLNMTRTPDVHFIAEARTEGTKFVVLSPDFSQIAKYCDEWIPIQAGQ
DTALWMAANHVILKEYYVDRQVPYFIDYVKRYTDLPFLVELEPNGTTYKTGRLLRARHVP
RYKDVENGDWKMLLLDANSGELRAPKGQVGDRWGSVHGKWNLSGEDTLDNSPLDPVLSFI
DRSDDVVQVGFDDFANGRIVSRGVPVRRIATDKGEILCATGFDIMMSQFGISRGLEGAFA
TSYDDEDAPYTPAWQERHTGIGRETAIRFAREFATTAEYTNGKSMVIVGASANHWYYNNL
CYRSATVALILCGCCGVNGGGINHYVGQEKLAPVAPWASIALALDWSKPPRVVQSSTWHY
AHSCQWRYEQEFTEYGLTAPNPRWAKGHAIDLEAKAVRSGWMPFTPHFNRNPIEVAAEAE
RAGAKSTEDIATHVIDQVASKKLNLAIEDPDAAENWPRLWFIWRGNAIQSSAKGHEFFLR
HYLGTHDNAIAEDRAKGKTHTVKYHDTAPRGKYDLVVDLNFRMDTSSLYSDIVLPTAFWY
EKNDLNTTDLHSFLHVLGQAVPPVWESKTDWDIFKLIAKKVSELAPLAFSKPVRDVVLQP
LMHDTPDELAQPEILDWAEGECKPVPGKSFPHVRVVERDYANLYNKFISFGPKAREDGVS
AVGVNVPIKKQYDQMLDNPIMPMPDPRHMRCVEWGGKRYPSLEDVLDGCNTVLLCAPEAN
GEVCYQAFHNEEHHVGLPLVDLAEPTRNVATTFYDLTRQPRRLLTSPCWTGMMNDGRAYS
AWCMNVERLVPWRTLTGRQTLYIDHQWYLDFGEHIPTYKPRLNPRKTGDIVKSRVDDRSL
VLNYITPHGKWNIHSTYKDNHRMLMLSRGMDPVWINDRDAEKVGIEDNDWVEVYNDNGVV
VTRANVSRRIQPGTCMYYHAVERTVYIPKS

>Nvulgaris_NxrA_OPH81928

mswildlvnp rerkweefyr nrwshdnvfr sthgvnctgg cswaiyvkdg iitwemqqtd

ypllerslpp yeprgcqrgi saswyvyspi rvkypyirgp lydlwkeaka shpdpvqawa

slvgdeqkrl riqkargkgg frrakwvelv eliaaaalyt arkwgpdrvm gfspipamsm

lsyaagsrfl qlfggvnmsf ydwyadlpts fpeiwgdqtd vcesadwyns kfivsmaanm

nmtrtpdvhf iaeartegtk fvvlspdfsq iakycdewip lqagqdtalw maanhvilke

yyidrqvpyf idyvkrytdl pflvelepng ntyktgrllr akrvaryqdv engdwkmlvl

dagtgepraf kgqvgdrwgs thgkwnlsae dtldnspidp vlsfidqsdg vvqvgfddfv

ngsvvsrgvp vkriatdkge vlvatgfdim msqfghsrgl egsfatsydd edapytpawq

erhtgigret airfarefat naeltngksm vivgasanhw yynnlcyrsa tvalilcgcc

gvnggginhy vgqeklapva pwntiamald wtkpprvvqs stwhyahscq wryeqeftey

gltapnprwa kghaidleak svrsgwmpft phfnrnpiel aaeaeragak stedivthvv

dqvaskkvnf aiddpdades wprmwfiwrg naiqssakgh efflrhylga hdnsiaedra

kgkthtvkyr dvaprgkydl vvdlnfrmnt tslysdivlp tafwyekndl nttdlhsflh

vlgqavppvw esktdweifk liskkvsela plafskpvkd vvvqplmhdt pdelaqpeil

dwaegeckpv pgksfphvrv verdyanlyn kfisfgpkar edgvsavgvn ipikkqydqm

ldnpvmpmpd arhmrcvewg gkrypsledv ldacntllmc apeangevcy qgfhneehhv

glplvdiaep trgvsstfyd ltrqprrilt spcwtgmtnd graysawcmn verlvpwrtl

tgrqslyldh qwyldfgehi ptykprlnpr ktgdivksrv ddrslvlnyi tphgkwnihs

tykdnhrmlm lsrgmdpvwi ndrdaekvgi edndwvevyn dngvvvtran vsrriqpgtc

myyhavertv yipksqerkw rggghnsltr trinplflag gyaqftygfn ywgptgiftr

dthvvvrkme klew

>Nmobilis_NxrA_WP_005004540

mgwiqdlinp ktrrweefyr nrwqhdnifr sthgvnctgg cswaiyvkdg iitwemqqtd

ypllgrgegg rgippyeprg cqrgisaswy vyspirvkyp ygkgvlldfw rearsshnnp

veawssivtd enkrkrwqka rgkggyrrtt wdellelias aclytaqkyg pdrvmgfspi

pamsmlsyaa gsrflqlfgg vnmsfydwya dlpnsfpeiw gdqtdvcesa dwynskfivs

masnlnmtrt pdvhfisear hegakfvvla pdfsqvskya dwwipvkkge dlglwmaagh

viytefyvkr qvpyfidyvt rytdmpflvk lekdgdgyrp gryltseevk kykkqenaaw

kqlvfdrksn earcpkgqig hrhgkhgqwn lkmedgldns piepvlsflg esddvamvqf

yefasqtvyk rgvpakkidt gsgsvlvatv ydlnmgqyav nrglpgdype syddlkpytp

awqeqfsgig rqtvirfare fagtaektkg rsmvivgasa nhwyhnnhiy raaincliac

gccgrngggm nhyvgqekla ivapwnalal agdwgikprl qqspvwhyvn sdswryegsf

eeyapsppna kwakghsvdl vaksvrmgwm phypqfnrsp levareaeka gakddkgmad

yvvqalkkkn lsfsvddpda penwprvwfi wrgnamqssa kgaefflrhy lgthdnavae

erakphvkhv kfrepaprgk fdlvvdinfr mdstalysdi vlptafwyek ndlnstdlhs

fihplgqavp pvwesksdwd ifkafakkis emapsvfsep fkdvvaaplt hdtpdeiaqr

dvkdwlegec epipgktmph frvverdysl lynkyislgs airengisgn gcsfpitkqy

deltnqpvgg spdprhrrav ewggkrypcv edaldaanvl lylapetnge vayqafkseq

ehcgvpltdl aepyrghqvt fydltrqprr llcspvwtgn cgdgraysaw tlqidrlvpf

rtltgrqhiy idhpwymdfg ehlctyrpkl dykkihdldn spiddktlil nyitphgkwn

ihstykdnhr mltlsrgmdp vwindkdaar vglkdndwve vyndngvivt ranvsrrvqs

gmclyyhave rtiyipksqi rggrragghn svtrtrinpv ylaggnaqft ylfnywgptg

imtrdthvav rkleklew

>Pstutzeri_NarH_AAZ43100

mkirsqvgmv lnldkcigch tcsitcknvw tsregmeyaw fnnvetkpgi gypkewenqd

kwkggwvrng dgtinpkigg kfrvlanifa npdlptiddy yepfdfdyqn lhtapisehq

pvarprslis gqrmekiewg pnweeilgte fakrrkdknf dkvqadiygq yentfmmylp

rlcehclnpa caascpsgai ykreedgivl idqekcrgwr mcisgcpykk iyfnwksgks

ekcifcfpri eagmptvcae tcvgrirylg vllydadrih evastvneqd lyakqleifl

dpfdpkvieq alndgvpmsv ieaaqkspvy kmavdwklal plhpeyrtlp mvwyvpplsp

iqnaasaghv smdgvlpdvd slriplryla nlltagdeep vklalkrmla mraykraeqv

dgvqdlkvle svglsvaqve dmyrylaian yedryvipta hreeamsdaf aersgcgfsf

gsgcsgasdt nmfgakkanr rdiiktvqlw ed
