# Supplementary material for: Differential Responses of the Catalytic Efficiency of Ammonia and Nitrite Oxidation to Changes in Temperature
Source: Front Microbiol. 2022 May 10;13:817986. doi: 10.3389/fmicb.2022.817986 (PMC9127996; doi:10.3389/fmicb.2022.817986)
Supplement: Supplementary file 1 [file Data_Sheet_1.zip › Supplemental Materials.DOCX]

Supplemental Materials

**Modeling the potential soil response of AOA, AOB, and NOB cultures.**

As stated in the methods section, we had to make several assumptions to compare Michaelis-Menten kinetics (MM) and Haldane (substrate-induced inhibition) parameters between pure cultures and soils. Pure cultures are calculated as µmol substrate mg protein^-1^ h^-1^ (eg. normalized to total protein) while soil rates are calculated as µmol substrate g soil^-1^ h^-1^ (eg. normalized to g soil used in the assay). We used mg protein as a proxy for cell density in pure cultures. If we assume that all of the activity from the soil is from one strain of AOB or NOB and use qPCR to "count cells" based on gene copy number then we can estimate the number of cells g soil^-1^. Finally, we can convert the number of cells g soil^-1^ to mg protein g soil^-1^ based on the relationship between cell volume and cell protein content. These assumptions make the rates calculated in pure cultures and soils have the same units: µmol substrate mg protein^-1^ h^-1^. Now we can calculate what the rate would be in the soil based on our qPCR measurement of "cell density": eg., if qPCR predicts 10000 cells g soil^-1^ and we calculate that 1000 cells = 1 mg protein then we can now predict the rate in a soil based on our pure culture model. Finally, using the MM and Haldane models we can make a prediction about the NO_2_^-^ +NO_3_^-^ accumulation rate or NO_2_^-^ oxidation rate (indicated by the lines in the figure) since we calculated the rate (µmol substrate mg protein^-1^ h^-1^) based on our pure culture data and cell density/protein estimate.

| Supplementary Table 1. Quantitative PCR protocols and primers | ***Nitrospira nxrB*** | *Nitrospira moscoviensis* | 95˚C, 5 min, 1x  95˚C, 40 sec, 40x  56.2˚C, 40 sec, 40x  72˚C, 90 sec, 40x  Melt curve starting at 65˚C | 10 µL Bio-rad SsoAdvanced Universal Sybr® Green Supermix  0.5µM forward primer, 0.5µM reverse primer  10 mg BSA ml^-1^, 10ng (5 µL 2.0 ng µL^-1^) template DNA  Nuclease free water to 20uL | nxrB169f: 5′- TACATGTGGT  GGAACA-3′ (Pester et al., 2014) | nxrB638r: 5′- CGGTTCTGG  TCRATCA-3′ (Pester et al., 2014) |
| --- | --- | --- | --- | --- | --- | --- |
|  | ***Nitrobacter nxrA*** | *Nitrobacter winogradskyi* | 95˚C, 5 min, 1x  94˚C, 30 sec, 40x  55˚C, 45 sec, 40x  72˚C, 45 sec, 40x  Melt curve starting at 65˚C |  | F1norA: 5′- CAGACCGACGTGTGCGAAAG-3′ (Poly et al., 2008) | F2843 R2 nxrA: 5′-TCCACAAGGAACGGAAGGTC-3′ (Wertz et al., 2008) |
|  | **AOB *amoA*** | *Nitrosospira multiformis* | 95˚C, 5 min, 1x  95˚C, 30 sec, 40x  60˚C, 45 sec, 40x  72˚C, 45 sec, 40x  Melt curve starting at 60˚C |  | amoA-1F 5’-GGGGTTTCTACTGGTGGT (Rotthauwe et al., 1997) | amoA-2R; 5’-CCCCTCKGSAAAGCCTTCTTC (K = G or T; S = G or C) (Rotthauwe et al., 1997) |
|  | **AOA *amoA*** | *Nitrososphaera viennensis* | 95˚C, 5 min, 1x  95˚C, 30 sec, 40x  55˚C, 30 sec, 40x  72˚C, 1 min, 40x  Melt curve starting at 55˚C |  | Arch-amoA-104F: 5′-GCAGGAGACTAYATHTTCTA-3′ (Alves et al., 2013) | Arch-amoA-616R: 5′-GCCATCCATC  TRTADGTCCA-3′ (Alves et al., 2013) |
|  |  | Standards | Thermocycler protocol | Reaction mix recipe | Forward primers | Reverse primers |

Supplemental Table 2. Gene copy numbers determined via BLAST search. *Genome unavailable.

| **Species** | **Gene** | **Copy #** | **Assumed gene copy #** |
| --- | --- | --- | --- |
| *Nph. viennensis* | amoA | 1 |  |
| *Nss. multiformis* | amoA | 3 |  |
| *Nm. europaea* | amoA | 2 |  |
| *Ns. moscoviensis* | nxrB | 5 |  |
| *Ns. japonica* | nxrB | 3 |  |
| *Nitrospira ND1** | nxrB |  | 3 |
| *Nb. winogradskyi* | nxrA | 2 |  |
| *Nb. hamburgensis* | nxrA | 3 |  |
| *Nb. vulgaris* | nxrA | 2 |  |

Supplemental Table 3. Physical properties of NH_3_ and NO_2_^-^ oxidizers used in this study. Cell dimensions are previously described in the literature (Lehtovirta-Morley et al. 2016, Urakawa et al. 2011, Watson et al. 1989). The relationship between cell volume and protein contents for *N. maritimus* (Urakawa, Martens-Habbena and Stahl 2011) were extrapolated to the other microorganisms.

|  | **Dimensions** | **Average cell volume** | **Protein/cell** |
| --- | --- | --- | --- |
| **Strain** | **(µm)** | **(um^3)** | **(fg)** |
| *Nitrosopumilus maritimus* | 0.25 x 0.5 - 0.9 | 0.0233 | 10 |
| *Nph. viennensis* | 0.6 - 0.8 | 0.19 | 82.6 |
| *Nss. multiformis* | 1.0 - 1.5 x 1.0 - 1.5 | 1.7 | 739.1 |
| *Nm. europaea* | 0.8 - 1.1 x 1.0 - 1.7 | 0.96 | 417.4 |
| *Nitrospira* | 0.3 - 0.4 x 0.8 - 1.0 | 0.09 | 39.1 |
| *Nitrobacter* | 0.5 - 0.8 x 1 - 2 | 0.6 | 260.9 |

Supplemental Table 4. qPCR results from soils used in this study. Lowercase letters indicate significant different between AOA and AOB amoA or NOB nxrA and nxrB within each soil (p ≤ 0.05). Capital letters indicate significant difference of gene copy numbers between soils (p ≤ 0.05).

|  |  | **Gene copies** |  |
| --- | --- | --- | --- |
| **Soil** | **Gene** | **Avg** | **Std Dev** |
| Columbia Basin | AOB amoA | 3.75E+06 ^aA^ | 4.02E+05 |
| Columbia Basin | AOA amoA | 3.13E+08 ^bB^ | 1.13E+07 |
| Columbia Basin | nxrA | 4.55E+07 ^aB^ | 6.35E+06 |
| Columbia Basin | nxrB | 8.74E+07 ^aA^ | 2.81E+07 |
| Coastal Plain | AOB amoA | 3.93E+06 ^aA^ | 2.00E+06 |
| Coastal Plain | AOA amoA | 1.70E+09 ^bA^ | 7.33E+08 |
| Coastal Plain | nxrA | 3.69E+08 ^aA^ | 1.20E+08 |
| Coastal Plain | nxrB | 5.74E+07 ^bA^ | 1.95E+07 |

Supplemental Tables 5. Kinetic parameters determined in temperature-controlled laboratory incubations of NH_3_ and NO_2_^-^ oxidizing cultures. Michaelis-Menten and Haldane parameters were calculated as describes in Materials and Methods. All values of *V_max_, K_m_, and K_i_* should be considered the apparent kinetic paramters since these were determined in whole cell incubations rather than with purified enzymes. The affinity constant (K_A_) and specific affinity (*a^o^_s_* ) were determined as described in D.K. Button 1998 and 1991. Lower case letters indicate where an ANOVA analysis indicated a significant difference in kinetic parameters between temperatures (p≤0.05).

|  | | *Nitrososphaera viennensis* | | | | | | | | | |
| --- | --- | --- | --- | --- | --- | --- | --- | --- | --- | --- | --- |
|  |  | | Michaelis Menten | | | Haldane | | |  |  |  |
| T (^o^C) | *V_max_†* | | | *K_m_†* | *V_max_/K_m_* | *V_max_†* | *K_m_†* | *K_i_** | *K_A_ (*µ*M)* | | *a^o^_s_* |
| 10 | 0.12 ^a^ (0.01) | | | 19.9 ^b^ (4.8) | 0.01 ^a^ (0.00) | 0.16 ^a^ (0.03) | 38.4^ab^ (15.3) | 1.2 (0.9) | 35.9 ^c^ (0.1) | 0.004 (0.001) | |
| 20 | 0.93 ^a^  (0.03) | | | 7.9 ^a^  (1.6) | 0.12 ^a^ (0.02) | 0.95 ^a^ (0.06) | 8.5 ^a^ (3.0) | 2.4 (91) | 24.0 ^b^ (0.5) | 0.058 (0.014) | |
| 30 | 8.10 ^b^ (0.29) | | | 20.3 ^b^ (3.2) | 0.40 ^a^ (0.05) | 10.16 ^b^ (0.89) | 35.2^ab^ (7.1) | 1.5 (0.6) | 20.3 ^a^ (1.8) | 0.402 (0.122) | |
| 42 | 10.33 ^c^ (0.64) | | | 6.1 ^a^ (3.5) | 2.21 ^b^ (1.39) | 17.14 ^c^ (2.53) | 30.3^ab^ (10.1) | 0.5 (0.2) | 42.8 ^d^ (0.5) | 0.424 (0.076) | |
| 50 | 8.14 ^b^ (0.64) | | | 13.8 ^a^ (5.4) | 0.65 ^ab^ (0.22) | 18.47 ^b^ (4.36) | 72.7 ^b^ (26.9) | 0.3 (0.1) | 72.9 ^e^ (1.4) | 0.188 (0.025) | |

*†Units of V_max_* µmol/mg protein/h*, units of K_m_* µM NH_3_ + NH_4_^+^, and *K_i_* units mM NH_3_ + NH_4_^+^; units of *a^o^_s_* liter mg cells^-1^ h^-1^

|  | | *Nitrosospira multiformis* | | | | | | | | | |
| --- | --- | --- | --- | --- | --- | --- | --- | --- | --- | --- | --- |
|  |  | | Michaelis Menten | | | Haldane | | | |  |  |
| T (^o^C) | *V_max_†* | | | *K_m_†* | *V_max_/K_m_* | *V_max_†* | *K_m_†* | *K_i_** | *K_A_ (*µ*M)* | | *a^o^_s_* |
| 10 | 3.0 ^a^  (0.1) | | | 73.7 ^a^ (4.4) | 0.04 ^a^ (0.00) | 3.0 ^a^ (0.2) | 73.7 ^a^ (9.0) | >1000 | 20.5 ^b^ (1.3) | | 0.064 (0.004) |
| 20 | 13.5 ^b^  (0.5) | | | 274.2 ^b^ (20.3) | 0.05 ^b^ (0.00) | 17.9 ^ab^ (3.9) | 395.1^ab^ (18.8) | 1.8 (1.5) | 105.7 ^d^ (6.5) | | 0.071 (0.004) |
| 30 | 24.6 ^c^  (1.2) | | | 332.0 ^b^ (32.1) | 0.07 ^c^ (0.00) | 24.6 ^ab^ (6.0) | 332.1^ab^ (108.2) | >1000 | 65.2 ^c^ (12.9) | | 0.124 (0.013) |
| 36 | 51.5 ^d^  (1.5) | | | 493.8 ^c^ (23.9) | 0.10 ^d^ (0.00) | 51.5 ^b^ (7.9) | 493.8^ab^ (94.4) | >1000 | 304.0 ^e^ (1.2) | | 0.129 (0.017) |
| 42 | 48.6 ^d^  (4.2) | | | 938.6 ^d^ (113.5) | 0.05 ^b^ (0.00) | 48.6 ^b^ (25.6) | 938.7 ^b^ (563.5) | >1000 | <0.01 ^a^ | | 0.132 (0.004) |

*†Units of V_max_* µmol/mg protein/h*, units of K_m_* µM NH_3_ + NH_4_^+^, and Ki units mM NH_3_ + NH_4_^+^; units of *a^o^_s_* liter mg cells^-1^ h^-1^; *p=1.0

|  | | *Nitrosomonas europaea* | | | | | | | | | |
| --- | --- | --- | --- | --- | --- | --- | --- | --- | --- | --- | --- |
|  |  | | Michaelis Menten | | | Haldane | | | |  |  |
| T (^o^C) | *V_max_†* | | | *K_m_†* | *V_max_/K_m_* | *V_max_†* | *K_m_†* | *K_i_** | *K_A_ (*µ*M)* | | *a^o^_s_* |
| 10 | 12.9^a^ (0.2) | | | 163.2^a^ (11.9) | 0.08 ^a^ (0.00) | 13.0^a^ (0.5) | 167.1^a^ (17.8) | 317 (1000) | 180.3 ^a^ (6.5) | | 0.075 (0.003) |
| 20 | 37.6^b^ (0.3) | | | 316.0^b^ (9.7) | 0.19 ^b^ (0.00) | 39.6^b^ (0.7) | 351.4^b^ (14.4) | 71.8 (23.2) | 305.3 ^c^ (4.2) | | 0.121 (0.002) |
| 30 | 71.6^c^ (0.9) | | | 439.1^c^ (18.7) | 0.16 ^c^ (0.00) | 75.3^c^ (2.6) | 482.1^c^ (35.2) | 82.3 (5.3) | 458.3 ^e^ (7.6) | | 0.160 (0.003) |
| 36 | 99.9^d^ (3.0) | | | 446.0^c^ (46.9) | 0.23 ^d^ (0.02) | 141.1^d^ (10.5) | 812.5^d^ (104.8) | 10.2 (2.5) | 405.0 ^d^ (38.2) | | 0.235 (0.006) |
| 42 | 128.7^e^ (1.6) | | | 571.4^d^ (23.8) | 0.23 ^d^ (0.00) | 140.6^d^  (5.2) | 669.0^e^ (47.3) | 47.8 (19.7) | 270.0 ^b^ (25.0) | | 0.279 (0.013) |

*†Units of V_max_* µmol/mg protein/h*, units of K_m_* µM NH_3_ + NH_4_^+^, and Ki units mM NH_3_ + NH_4_^+^; *p=0.928

|  | | *Nitrospira moscoviensis* | | | | | | | | |
| --- | --- | --- | --- | --- | --- | --- | --- | --- | --- | --- |
|  |  | | Michaelis Menten | | | Haldane | | |  |  |
| T (^o^C) | *V_max_†* | | | *K_m_†* | *V_max_/K_m_* | *V_max_†* | *K_m_†* | *K_i_* | *K_A_ (*µ*M)* | *a^o^_s_* |
| 10 | 6.4 ^a^ (0.4) | | | 1.8 ^a^ (0.8) | 3.9 ^a^ (1.5) | 34.7 (140) | 34.8 (170) | 0.007 (0.0) | 6.0 ^c^ (0.3) | 1.66 (0.19) |
| 20 | 12.9 ^b^ (0.2) | | | 1.1 ^a^ (0.2) | 12.6 ^b^ (2.5) | 13.9 ^a^ (0.4) | 1.7 ^a^ (0.4) | 1.3 ^a^ (0.3) | 2.3 ^a^ (0.2) | 7.55 (1.18) |
| 30 | 38.6 ^c^ (0.9) | | | 11.3 ^b^ (1.2) | 3.4 ^a^ (0.3) | 45.8 ^b^ (2.4) | 16.5 ^b^ (2.4) | 0.8 ^b^ (0.2) | 3.6 ^b^ (0.9) | 5.16 (0.33) |
| 40 | 61.2 ^d^ (1.8) | | | 18.5 ^c^ (2.2) | 3.3 ^a^ (0.3) | 77.8 ^c^ (3.7) | 30.1 ^c^ (3.4) | 0.8 ^b^ (0.1) | 26.0 ^d^ (0.9) | 2.75 (0.04) |
| 50 | 58.8 ^d^ (2.5) | | | 13.6 ^b^ (2.6) | 4.4 ^a^ (0.7) | 74.8 ^c^ (5.6) | 21.8 ^b^ (4.2) | 0.6 ^b^ (0.2) | 32.6 ^e^ (0.6) | 2.54 (0.33) |

*†Units of V_max_* µmol/mg protein/h*, units of K_m_* µM, and K_i_ units mM; Haldane 10^o^C data excluded from ANOVA analysis

|  | | *Nitrospira japonica strain NJ1* | | | | | | | | |
| --- | --- | --- | --- | --- | --- | --- | --- | --- | --- | --- |
|  |  | | Michaelis Menten | | | Haldane | | |  |  |
| T (^o^C) | *V_max_†* | | | *K_m_†* | *V_max_/K_m_* | *V_max_†* | *K_m_†** | *K_i_** | *K_A_ (*µ*M)* | *a^o^_s_* |
| 10 | 8.9 ^a^ (0.7) | | | 13.2 ^a^ (5.1) | 0.73 (0.25) | 24.6 ^a^ (6.1) | 88.4 (33.1) | 0.2 (0.1) | 25.2 ^b^ (2.1) | 0.463 (0.029) |
| 20 | 25.3 ^b^ (1.1) | | | 50.6 ^b^ (6.9) | 0.50 (0.05) | 29.4 ^a^ (4.2) | 66.9 (18.3) | 2.7 (2.6) | 31.1 ^bc^ (3.4) | 0.619 (0.034) |
| 30 | 23.9 ^b^ (1.3) | | | 30.1 ^a^ (6.3) | 0.81 (0.13) | 32.4 ^a^ (5.7) | 56.3 (19.6) | 1.1 (0.7) | 16.5 ^a^ (3.9) | 0.755 (0.024) |
| 37 | 36.8 ^c^ (2.0) | | | 59.7 ^c^ (9.9) | 0.62 (0.07) | 49.5 ^ab^ (11.1) | 101.1 (37.9) | 1.4 (1.2) | 35.5 ^c^ (4.8) | 0.773 (0.024) |
| 42 | 35.4 ^c^ (2.0) | | | 40.5 ^b^ (7.8) | 0.89 (0.13) | 62.7 ^b^ (14.6) | 111.7 (41.3) | 0.6 (0.3) | 55.6 ^d^ (2.4) | 0.736 (0.024) |

*†Units of V_max_* µmol/mg protein/h*, units of K_m_* µM, and Ki units mM; *p≥0.245

|  | | *Nitrospira ND1* | | | | | | | | |  |
| --- | --- | --- | --- | --- | --- | --- | --- | --- | --- | --- | --- |
|  |  | | Michaelis Menten | | | Haldane | | |  |  |  |
| T (^o^C) | *V_max_†* | | | *K_m_†** | *V_max_/K_m_* | *V_max_†** | *K_m_†** | *K_i_** | *K_A_ (*µ*M)* | *a^o^_s_* | |
| 10 | 7.2 ^a^  (0.5) | | | 13.9 ^a^ (4.7) | 0.56 ^bc^ (0.16) | 7.2 (1.0) | 13.9 (6.6) | >1000 | 19.0 ^c^ (2.5) | 0.436 (0.046) | |
| 20 | 20.6 ^b^  (1.2) | | | 50.4 ^a^ (9.4) | 0.42 ^b^ (0.05) | 20.6 (3.4) | 50.4 (18.2) | >1000 | 9.7 ^b^ (5.9) | 0.684 (0.023) | |
| 30 | 24.5 ^b^  (0.9) | | | 38.1 ^a^ (4.7) | 0.65 ^c^ (0.06) | 25.7 (2.4) | 42.2 (9.3) | 8.9 (17.4) | 20.4 ^c^ (2.6) | 0.837 (0.023) | |
| 37 | 24.7^b^  (0.9) | | | 84.1 ^b^ (8.7) | 0.30 ^a^ (0.02) | 4.1 (3.0) | 154.0 (174.6) | >1000 | 117.7 ^d^ (1.5) | 0.244 (0.008) | |
| 42 | 10.7 ^a^  (5.0) | | | 505.9^¶^ (395.4) | 0.03 ^a^ (0.02) | 10.7 (24.9) | >500 (1000) | >1000 | <<0.01 ^a^ | 0.068 (0.014) | |

*†Units of V_max_* µmol/mg protein/h*, units of K_m_* µM, and Ki units mM; *p≥0.181; ^¶^ 42^o^C *K_m_* value omitted from one-way ANOVA analysis

|  | | *Nitrobacter winogradskyi* | | | | | | | | |  |
| --- | --- | --- | --- | --- | --- | --- | --- | --- | --- | --- | --- |
|  |  | | Michaelis Menten | | | Haldane | | |  |  |  |
| T (^o^C) | *V_max_†* | | | *K_m_†* | *V_max_/K_m_* | *V_max_†** | *K_m_†** | *K_i_** | *K_A_ (*µ*M)* | *a^o^_s_* | |
| 10 | 12.9 ^a^  (0.5) | | | 110.0 ^a^ (13.8) | 0.12 ^a^ (0.01) | 18.6  (3.5) | 194.0 (55.1) | 1.4 (0.8) | 134.2 ^a^ (4.5) | 0.106 (0.004) | |
| 20 | 16.0 ^a^  (0.9) | | | 102.1 ^a^ (18.7) | 0.16 ^b^ (0.02) | 45.6 (19.2) | 429.9 (225.9) | 0.3 (0.2) | 225.5 ^b^ (0.5) | 0.098 (0.004) | |
| 30 | 34.2 ^b^  (1.6) | | | 302.2 ^b^ (46.7) | 0.11 ^a^ (0.01) | 36.4  (5.4) | 336.3 (99.7) | 30.8 (70.0) | 488.0 ^c^ (10.0) | 0.086 (0.007) | |
| 37 | 33.6 ^b^  (3.1) | | | 416.5 ^b^ (110.9) | 0.08 ^a^ (0.02) | 33.5  (9.8) | 416.5 (232.2) | >1000 | 1188.3 ^d^ (37.5) | 0.042 (0.003) | |

*†Units of V_max_* µmol/mg protein/h*, units of K_m_* µM, and Ki units mM; *p≥0.10

|  | | *Nitrobacter hamburgensis* | | | | | | | | | | | |  |
| --- | --- | --- | --- | --- | --- | --- | --- | --- | --- | --- | --- | --- | --- | --- |
|  |  | | Michaelis Menten | | | | Haldane | | | | |  |  |  |
| T (^o^C) | *V_max_†* | | | *K_m_†* | *V_max_/K_m_* | *V_max_†** | | *K_m_†** | | *K_i_** | *K_A_ (*µ*M)* | | *a^o^_s_* | |
| 10 | 31.0 ^a^ (2.5) | | | 257.0 ^a^ (80.0) | 0.13 ^c^ (0.03) | 43.7 (12.8) | | 535.8 (300) | 7.2 (6.8) | | 472.2 ^a^ (21.5) | | 0.085 (0.005) | |
| 20 | 43.2 ^b^ (1.8) | | | 450.7^ab^ (62.2) | 0.10 ^bc^ (0.01) | 43.4 (5.3) | | 449.9 (100) | 82.0 (2e7) | | 581.1 ^b^ (19.0) | | 0.084 (0.003) | |
| 30 | 64.8^c^ (4.2) | | | 769.6 ^b^ (137.8) | 0.09 ^b^ (0.01) | 303.0 (215.9) | | 1334.1 (450) | 1.0 (0.9) | | 1269.7 ^c^ (5.5) | | 0.064 (0.003) | |
| 35 | 66.9 ^c^ (4.0) | | | 1303.0^c^ (180.3) | 0.05 ^a^ (0.00) | 203.8 (135.6) | | 1640.8 (410) | 2.0 (1.9) | | 1358.3 ^d^ (17.6) | | 0.005 (0.001) | |
| 42 | 61.1 ^c^ (5.1) | | | 1662.8^c^ (302.8) | 0.04 ^a^ (0.00) | 23962.9 (2e6) | | 914.0 (1e8) | 11.1 (1.3) | | 2345.0 ^e^ (27.8) | | 0.024 (0.002) | |

*†Units of V_max_* µmol/mg protein/h*, units of K_m_* µM, and Ki units mM; *p=1.0

|  | | *Nitrobacter vulgaris* | | | | | | | | | | |  |
| --- | --- | --- | --- | --- | --- | --- | --- | --- | --- | --- | --- | --- | --- |
|  |  | | Michaelis Menten | | | | Haldane | | | |  |  |  |
| T (^o^C) | *V_max_* | | | *K_m_* | *V_max_/K_m_* | *V_max_** | | *K_m_** | *K_i_** | *K_A_ (*µ*M)* | | *a^o^_s_* | |
| 10 | 45.6 ^a^  (2.8) | | | 30.6 ^a^ (8.1) | 1.55 ^b^ (0.33) | 72.8 (15.0) | | 77.2 (29.5) | 0.9 (0.5) | 37.9 ^a^ (3.8) | | 1.33 (0.03) | |
| 20 | 67.1 ^b^  (2.5) | | | 37.8 ^a^ (5.8) | 1.80 ^c^ (0.21) | 85.4 (9.0) | | 60.9 (13.4) | 2.2 (1.0) | 59.3 ^ab^ (2.0) | | 1.39 (0.04) | |
| 30 | 100.4 ^c^ (2.0) | | | 83.7 ^b^ (5.7) | 1.20 ^b^ (0.06) | 100.4 (5.9) | | 83.7 (10.4) | >1000 | 84.8 ^b^ (2.5) | | 1.19 (0.02) | |
| 35 | 94.6 ^c^ (10.3) | | | 291.6 ^c^ (78.7) | 0.33 ^a^ (0.06) | 94.6 (40.4) | | 291.6 (195.1) | >1000 | 228.2 ^d^ (23.5) | | 0.36 (0.01) | |
| 42 | 102.4 ^c^ (7.6) | | | 210.1 ^c^ (42.8) | 0.50 ^a^ (0.07) | 183.6 (86.1) | | 475.0 (299.0) | 1.1 (1.1) | 125.1 ^c^ (18.1) | | 0.61 (0.06) | |

*†Units of V_max_* µmol/mg protein/h*, units of K_m_* µM, and Ki units mM; *p≥0.05

Supplemental Tables 6. Comparison of the substrate affinity of NH_3_ oxidizing cultures in response to changing NH_3_/NH_4_^+^ distribution with temperature. Concentrations of NH_3_ were determined as described by Groeneweg et al. (1994). Michaelis-Menten and Haldane parameters were calculated as describes in Materials and Methods. The affinity constant (K_A_) and specific affinity (*a^o^_s_* ) were determined as described previously (Button 1985, Button 1998). Lower case letters indicate where an ANOVA analysis indicated a significant difference in kinetic parameters between temperatures (p≤0.05).

| *Nitrososphaera viennensis* | | | | | | | | | | | | |  |  |
| --- | --- | --- | --- | --- | --- | --- | --- | --- | --- | --- | --- | --- | --- | --- |
|  | Michaelis Menten | | Haldane | |  | | Specific affinity | | | |  | | | |
| T (^o^C) | *K_m_*  µM (NH_3_+NH_4_^+^) | *K_m_*  µM (NH_3_) | *K_m_*  µM (NH_3_+NH_4_^+^) | *K_m_*  µM (NH_3_) | | *K_A_*  µM (NH_3_+NH_4_^+^) | | *K_A_*  µM (NH_3_) | *a^o^_s_*  l mg^-1^ h^-1^  (NH_3_+NH_4_^+^) | | | *a^o^_s_*  l mg^-1^ h^-1^  (NH_3_) | |  |
| 10 | 19.9 ^b^ (4.8) | 0.4 ^a^ (0.1) | 38.4^ab^ (15.3) | 0.7 ^a^ (0.3) | | 35.9 ^c^ (0.1) | | 0.7 ^a^ (0.0) | | 0.004 (0.001) | | 0.18 (0.01) | |  |
| 20 | 7.9 ^a^  (1.6) | 0.3 ^ac^ (0.1) | 8.5 ^a^ (3.0) | 0.3 ^a^ (0.1) | | 24.0 ^b^ (0.5) | | 0.9 ^b^ (0.0) | | 0.058 (0.014) | | 1.51 (0.04) | |  |
| 30 | 20.3 ^b^ (3.2) | 1.5 ^b^ (0.2) | 35.2^ab^ (7.1) | 2.6 ^b^ (0.5) | | 20.3 ^a^ (1.8) | | 1.5 ^c^ (0.1) | | 0.402 (0.122) | | 7.69 (1.64) | |  |
| 42 | 6.1 ^a^ (3.5) | 0.9 ^c^ (0.5) | 30.3^ab^ (10.1) | 4.6 ^c^ (1.5) | | 42.8 ^d^ (0.5) | | 6.4 ^d^ (0.1) | | 0.424 (0.076) | | 2.78 (0.06) | |  |
| 50 | 13.8 ^a^ (5.4) | 3.1 ^d^  (1.2) | 72.7 ^b^ (26.9) | 16.4^d^ (5.1) | | 72.9 ^e^ (1.4) | | 16.4^e^ (0.3) | | 0.188 (0.025) | | 0.85 (0.13) | |  |

|  | | *Nitrosospira multiformis* | | | | | | | | | | | | | | |
| --- | --- | --- | --- | --- | --- | --- | --- | --- | --- | --- | --- | --- | --- | --- | --- | --- |
|  | Michaelis Menten | | | Haldane | |  | | Specific affinity | | | |  | | |  |  |
| T (^o^C) | *K_m_*  µM (NH_3_+NH_4_^+^) | | *K_m_*  µM (NH_3_) | *K_m_*  µM (NH_3_+NH_4_^+^) | *K_m_*  µM (NH_3_) | | *K_A_*  µM (NH_3_+NH_4_^+^) | | *K_A_*  µM (NH_3_) | *a^o^_s_*  l mg^-1^ h^-1^  (NH_3_+NH_4_^+^) | | | | *a^o^_s_*  l mg^-1^ h^-1^  (NH_3_) | |  |
| 10 | 73.7 ^a^ (4.4) | | 1.4 ^a^ (0.1) | 73.7 ^a^ (9.0) | 1.4 ^a^ (0.2) | | 20.5 ^b^ (1.3) | | 0.4 ^a^ (0.0) | | 0.064 (0.004) | | 2.86 (0.24) | | | |
| 20 | 274.2 ^b^ (20.3) | | 10.5 ^ab^ (0.8) | 395.1^ab^ (1.8.8) | 15.1 ^ab^ (0.7) | | 105.7 ^d^ (6.5) | | 4.0 ^b^ (0.3) | | 0.071 (0.004) | | 1.46 (0.12) | | | |
| 30 | 332.0 ^b^ (32.1) | | 24.7 ^b^ (2.4) | 332.1^ab^ (108.2) | 24.7 ^b^ (8.1) | | 65.2 ^c^ (12.9) | | 4.9 ^b^ (0.9) | | 0.124 (0.013) | | 1.43 (0.07) | | | |
| 36 | 493.8 ^c^ (23.9) | | 53.1 ^c^ (2.6) | 493.8^ab^ (94.4) | 53.1 ^c^ (10.1) | | 304.0 ^e^ (1.2) | | 32.7 ^c^ (0.1) | | 0.129 (0.017) | | 1.27 (0.20) | | | |
| 42 | 938.6 ^d^ (113.5) | | 141.3 ^d^  (17.1) | 938.7 ^b^ (563.5) | 141.3 ^d^ (84.5) | | <0.01 ^a^ | | 0.001 ^d^ | | 0.132 (0.004) | | 0.87 (0.05) | | | |

| *Nitrosomonas europaea* | | | | | | | | | | | | | |
| --- | --- | --- | --- | --- | --- | --- | --- | --- | --- | --- | --- | --- | --- |
|  | Michaelis Menten | | Haldane | |  | | Specific affinity | | | |  | |  |
| T (^o^C) | *K_m_*  µM (NH_3_+NH_4_^+^) | *K_m_*  µM (NH_3_) | *K_m_*  µM (NH_3_+NH_4_^+^) | *K_m_*  µM (NH_3_) | | *K_A_*  µM (NH_3_+NH_4_^+^) | | *K_A_*  µM (NH_3_) | *a^o^_s_*  l mg^-1^ h^-1^  (NH_3_+NH_4_^+^) | | | *a^o^_s_*  l mg^-1^ h^-1^  (NH_3_) | |
| 10 | 163.2^a^ (11.9) | 2.9 ^a^ (0.2) | 167.1^a^ (17.8) | 3.1 ^a^ (0.3) | | 180.3 ^a^ (6.5) | | 3.3 ^a^ (0.1) | | 0.075 (0.003) | | 4.13 (0.18) | |
| 20 | 316.0^b^ (9.7) | 12.1 ^a^ (0.4) | 351.4^b^ (14.4) | 13.4 ^a^ (0.5) | | 305.3 ^c^ (4.2) | | 11.7 ^a^ (0.2) | | 0.121 (0.002) | | 3.18 (0.08) | |
| 30 | 439.1^c^ (18.7) | 32.7 ^b^ (1.4) | 482.1^c^ (35.2) | 35.9 ^b^ (2.6) | | 458.3 ^e^ (7.6) | | 34.1 ^b^ (0.6) | | 0.160 (0.003) | | 2.15 (0.05) | |
| 36 | 446.0^c^ (46.9) | 47.9 ^c^ (5.0) | 812.5^d^ (104.8) | 87.3 ^b^ (11.3) | | 405.0 ^d^ (38.2) | | 43.5 ^c^ (4.1) | | 0.235 (0.006) | | 1.92 (0.05) | |
| 42 | 571.4^d^ (23.8) | 86.0 ^d^  (3.6) | 669.0^e^ (47.3) | 100.7 ^c^ (7.1) | | 270.0 ^b^ (25.0) | | 40.7 ^d^ (3.8) | | 0.279 (0.013) | | 1.55 (0.05) | |

Supplemental Table 7. Results of the regression analysis comparing Michaelis-Menten (MM) and Haldane (H) kinetic models with rates of NH_3_ and NO_2_^-^ oxidation measured in Tillamook and Columbia Basin soils with and without added NH_4_^+^. The effects of Haldane inhibition were not determined (n.d.) in the Tillamook soils because NO_2_^-^ did not accumulate.

| Nitrifying organism | Tillamook | | | | | | Columbia Basin | | | | | |
| --- | --- | --- | --- | --- | --- | --- | --- | --- | --- | --- | --- | --- |
|  | No added N | | Added NH_4_^+^ | | | | No added N | | Added NH_4_^+^ | | | |
|  | MM | MM | MM | MM | H | H | MM | MM | MM | MM | H | H |
|  | R^2^ | *p* | R^2^ | *p* | R^2^ | *p* | R^2^ | *p* | R^2^ | *p* | R^2^ | *p* |
| Nph. viennensis | **0.815** | **0.001** | **0.671** | **0.007** | **0.685** | **0.006** | 0.014 | 0.713 | **0.506** | **0.010** | **0.918** | **0.000** |
| Nss. multiformis | 0.001 | 0.931 | 0.029 | 0.659 | 0.025 | 0.683 | 0.000 | 0.987 | 0.001 | 0.930 | 0.000 | 0.969 |
| Nm.  europaea | 0.001 | 0.931 | 0.014 | 0.758 | 0.013 | 0.773 | 0.000 | 0.987 | 0.045 | 0.507 | 0.288 | 0.072 |
| Ns. moscoviensis | 0.146 | 0.309 | 0.323 | 0.110 | n.d. | n.d. | 0.018 | 0.681 | 0.037 | 0.550 | 0.056 | 0.461 |
| Ns.  japonica | 0.034 | 0.635 | 0.203 | 0.224 | n.d. | n.d. | 0.077 | 0.381 | 0.063 | 0.430 | 0.035 | 0.558 |
| Nitrospira ND1* | 0.034 | 0.635 | 0.099 | 0.409 | n.d. | n.d. | 0.077 | 0.381 | **0.361** | **0.039** | 0.200 | 0.145 |
| Nb. winogradskyi | 0.146 | 0.309 | 0.063 | 0.516 | n.d. | n.d. | 0.077 | 0.381 | **0.688** | **0.001** | **0.591** | **0.004** |
| Nb. hamburgensis | 0.340 | 0.099 | 0.250 | 0.519 | n.d. | n.d. | 0.077 | 0.381 | **0.825** | **0.000** | **0.856** | **0.000** |
| Nb.  vulgaris | **0.641** | **0.010** | 0.000 | 0.955 | n.d. | n.d. | 0.015 | 0.703 | **0.648** | **0.002** | **0.687** | **0.001** |

**References**

Button DK. 1985. Kinetics of Nutrient-Limited Transport and Microbial-Growth. Microbiol Rev.49:270-297.

Button DK. 1998. Nutrient uptake by microorganisms according to kinetic parameters from theory as related to cytoarchitecture. Microbiol Mol Biol Rev. Sep;62:636-+.

Groeneweg J, Sellner B, Tappe W. 1994. Ammonia oxidation in *Nitrosomonas* at NH_3_ concentrations near K_m_ - Effects of pH and temperature. Water Research. Dec;28:2561-2566.

Lehtovirta-Morley LE, Sayavedra-Soto LA, Gallois N, Schouten S, Stein LY, Prosser JI, Nicol GW. 2016. Identifying potential mechanisms enabling acidophily in the ammonia-oxidising archaeon 'Candidatus Nitrosotalea devanaterra'. Appl Environ Microbiol. Feb 19.

Urakawa H, Martens-Habbena W, Stahl DA. 2011. Physiology and genomics of ammonia-oxidizing *Archaea*. In: Nitrification. Washington D.C.: ASM Press. p. 117 - 155.

Watson SW, Bock E, Harms H, Koops HP, Hooper A. 1989. Nitrifying bacteria. In: Bergy’s Manual of Systemic Bacteriology. Baltimore, MD: The Williams & Williams Co. p. 1808 - 1834.
